# Unravelling the Roles of Integral Polypeptides in Excitation Energy Transfer of Photosynthetic RC-LH1 Supercomplexes

**DOI:** 10.1021/acs.jpcb.3c04466

**Published:** 2023-08-09

**Authors:** Owen Thwaites, Bern M. Christianson, Alexander J. Cowan, Frank Jäckel, Lu-Ning Liu, Adrian M. Gardner

**Affiliations:** † Stephenson Institute of Renewable Energy, University of Liverpool, Liverpool L69 7ZF, U.K.; ‡ Department of Physics, University of Liverpool, Liverpool L69 7ZE, U.K.; § Institute of Systems, Molecular and Integrative Biology, University of Liverpool, Liverpool L69 7ZB, U.K.; ∥ Department of Chemistry, University of Liverpool, Liverpool L69 7ZD, U.K.; ⊥ College of Marine Life Sciences, and Frontiers Science Center for Deep Ocean Multispheres and Earth System, Ocean University of China, Qingdao 266003, China; # Early Career Laser Laboratory, University of Liverpool, Liverpool L69 3BX, U.K.

## Abstract

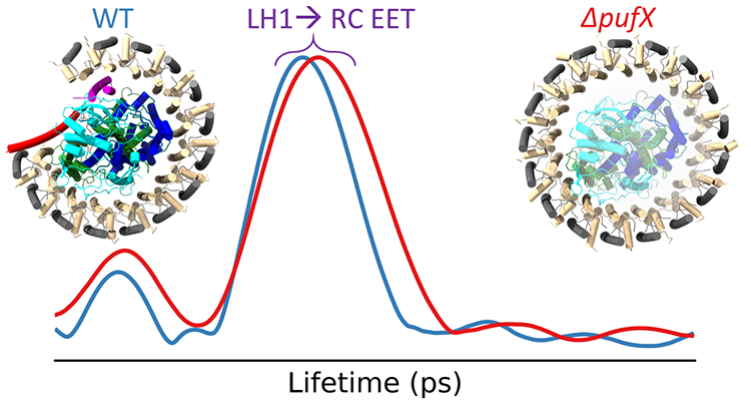

Elucidating the photosynthetic processes that occur within the reaction center-light-harvesting 1 (RC-LH1) supercomplexes from purple bacteria is crucial for uncovering the assembly and functional mechanisms of natural photosynthetic systems and underpinning the development of artificial photosynthesis. Here, we examined excitation energy transfer of various RC-LH1 supercomplexes of *Rhodobacter sphaeroides* using transient absorption spectroscopy, coupled with lifetime density analysis, and studied the roles of the integral transmembrane polypeptides, PufX and PufY, in energy transfer within the RC-LH1 core complex. Our results show that the absence of PufX increases both the LH1 → RC excitation energy transfer lifetime and distribution due to the role of PufX in defining the interaction and orientation of the RC within the LH1 ring. While the absence of PufY leads to the conformational shift of several LH1 subunits toward the RC, it does not result in a marked change in the excitation energy transfer lifetime.

## Introduction

Strategies that harness solar energy to produce high-energy fuels are being urgently sought to provide alternatives to fossil fuels, which have a significant negative impact on the environment.^1^ Since the dawn of life, photosynthesis has been used by nature to convert solar energy into chemical energy, which is essential for the survival and sustenance of life on earth.^2,3^ The photosynthetic systems of purple bacteria provide a model for exploring the photodynamic steps required for trapping sunlight and converting it to a useful fuel. In purple phototropic bacteria, the essential photosynthetic unit is composed of a reaction center (RC) encircled by light-harvesting complex 1 (LH1) forming the RC-LH1 core supercomplex, which utilizes energy from sunlight to drive the primary redox reactions of anoxygenic photosynthesis.^4^ Understanding the structures and energy transfer of natural RC-LH1 complexes is paramount in uncovering the mechanisms of anoxygenic photosynthesis and underpinning the development of artificial photosynthesis.

**Figure 1:**
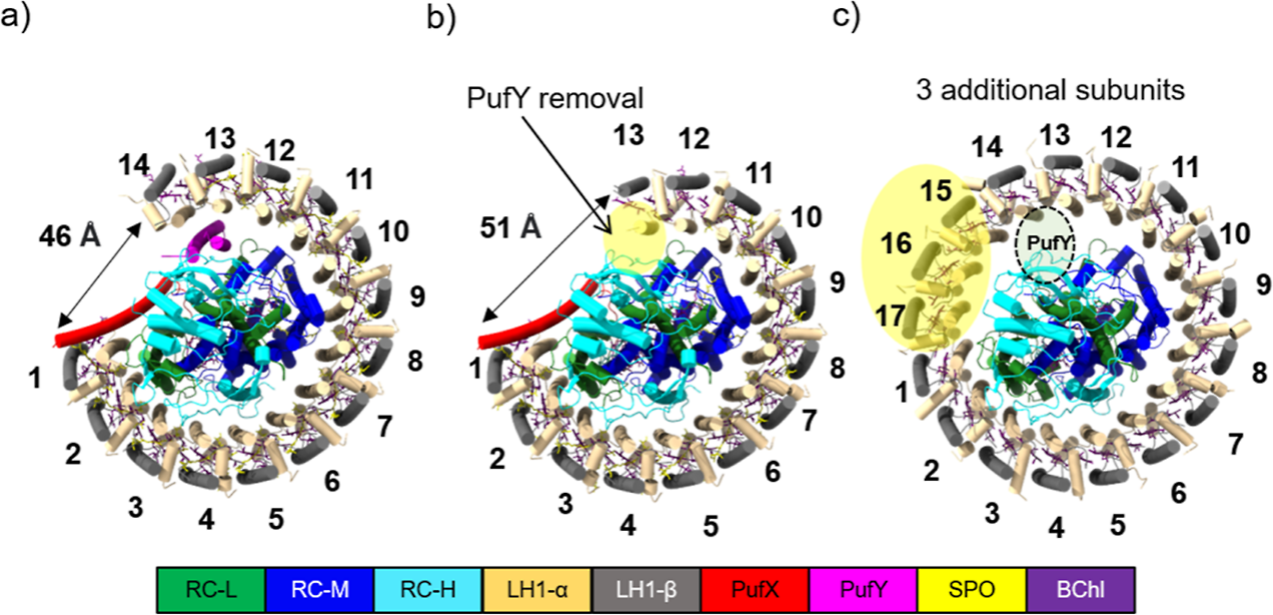
Cryo-EM structures of the RC-LH1 monomers from *Rba. sphaeroides* viewed from the cytoplasmic side. (a) WT monomer (PDB ID: 7VNY), (b) Δ*pufY* monomer (PDB ID: 7VNM), and (c) Δ*pufX* monomer (PDB ID: 7VOY). The yellow circles highlight the structural differences of the Δ*pufY* monomer and Δ*pufX* monomer compared to that of the WT RC-LH1 monomer.

High-resolution cryo-electron microscopy (cryo-EM) structures of the RC-LH1 supercomplexes of the model purple bacterium *Rhodobacter* (*Rba.*) *sphaeroides* 2.4.1 have been recently obtained.^5^ The wild-type (WT) *Rba. sphaeroides* RC-LH1 complexes form both monomers and dimers in cells, comprising LH1 α and β-subunits, RC H, L, and M subunits, as well as the PufX and PufY (the latter was also named protein-Y or protein-U) transmembrane (TM) polypeptides. The monomeric RC-LH1 consists of one RC surrounded by an LH1 ring of 14 αβ-heterodimers with a large gap adjacent to PufX and PufY (Figure 1a); the RC-LH1 dimer contains a continuous S-shaped array of LH1 αβ-subunits surrounding two RCs, with two copies of PufX located in the center, mediating the dimerization of two C-shaped monomers (Figure S1a,b). The PufX peptide is made up of 82 amino acids and has three parts: a short N-terminal tail, a central transmembrane helix, and a C-terminal loop. The C-terminal loop links to the RC-L subunit on the periplasmic side of RC-LH1, while the N-terminal tail is near the first LH1 subunit on the cytoplasmic side; the transmembrane helix is positioned diagonally to the membrane plane. This unique arrangement of PufX results in a gap within the LH1 ring that prevents LH1 subunits from fully encircling the RC, which is important for rapid quinone exchange.^5–9^ Deletion of PufX (denoted Δ*puf*X) led to only monomeric RC-LH1 supercomplexes to be formed, which consist of fully closed LH1 ring of 17 αβ-subunits around the RC, thereby resulting in a loss of efficient phototrophic growth of the cells.^9–12^ The PufY peptide is located between the RC and the LH1-13 and LH1-14 subunits near the opening on the opposite side of PufX (Figure 1a). Deleting PufY (denoted Δ*puf*Y) resulted in the production of both monomeric and dimeric RC-LH1 complexes, in which the final LH1 αβ-pair(s) is lost, forming a larger gap within the LH1 ring (Figures 1b and S1c,d),^5,13^ indicating the role of PufY in stabilizing the LH1 ring which has potential implications for quinone diffusion to the RC. These RC-LH1 variants provide a paradigm for exploring the assembly and functional principles of the RC-LH1 core supercomplex.

Each LH1 αβ-heterodimer of *Rba. sphaeroides* sandwiches two carotenoids and two bacteriochlorophyll (BChl) *a* chromophores (referred to as ^LH1^BChl), which are responsible for the ∼880 nm absorption assigned to the S_1_ ← S_0_ electronic transition,^13^ denoted ^LH1^BChl(Q_y_). The RC contains a special pair of BChl *a* (^RC^P), a single carotenoid, and two branches (A and B) with a pseudo twofold symmetry, each containing one BChl *a* monomer, (^RC^BChl), one bacteriopheophytin (^RC^BPhe), and one quinone (Q). Upon absorption of ∼880 nm light by ^LH1^BChls, energy is transferred to ^RC^P through excitation energy transfer (EET) with a typical lifetime of ∼40 ps.^13–17^ Subsequent rapid (∼3 ps) electron transfer (ET) forms a charge-separated P^+^BChl_A_^–^ singlet state, which undergoes further ultrafast (<1 ps) ET to produce P^+^BPhe_A_^–^.^17^ Initial charge separation occurs with near unity yield via the A-branch. Slower ET processes involve reducing Q_A_ (τ ≈ 200 ps) and finally Q_B_ (τ ≈ 100 μs).^18,19^ The photoxidized P^+^ is reduced by cytochrome (Cyt) *c*^2+^, and the process repeats, resulting in the formation of a doubly reduced Q_B_ ligand (QH_2_, quinol), which leaves the RC-LH1 supercomplex and is then oxidized by neighboring Cyt *bc*_1_.^10,20,21^

Despite advances in understanding the structure of *Rba. sphaeroides* RC-LH1, how the integral TM proteins, PufX and PufY, determine the EET process of the RC-LH1 core complex, which is crucial for initial energy conversion, remains unclear. In this work, we probe the picosecond–nanosecond (ps–ns) photodynamics of the WT, Δ*pufY*, Δ*pufX*, and Δ*pufXY* (in which both *pufX* and *pufY* genes were deleted, and like the Δ*pufX* supercomplex, exists in only monomeric form, Figure S2) RC-LH1 supercomplexes from *Rba. sphaeroides* by using transient absorption (TA) spectroscopy.

## Methods

Genomic deletions of *pufX* were constructed in WT *Rba. sphaeroides* using the allelic exchange suicide vector pk18mobsacB using the previously described method.^5,22^ Deletion of *pufX* (rsp_0255) was performed as follows: primer pairs XupF and XupR (AGTCTCTAGAGCACCTATCTCCGCGCAG and CTGCCCCGAGACTTGTCTCAGTGTGATCGCTCCTCAGTTCAG) and XdownF and XdownR (CTGAACTGAGGAGCGATCACACTGAGACAAGTCTCGGGGCAG and ATGCAAGCTTGTCGTAGGCGGATTCCGAGC) were used to amplify the regions flanking rsp_0255, fused via PCR, digested, and cloned into the *Bam*HI and *Hin*dIII sites of pk18mobsacB. Regions flanking *pufY* (rsp_7571) could not be fused via the same method, so a 1-kbp synthesized DNA fragment (Genewiz, Germany), comprising two 500-bp regions identical to the up- and downstream DNA sequences flanking rsp_7571, was digested and cloned into the same sites of the pK18mobsacB vector.

The resulting plasmids were transferred from *Escherichia* (*E.*) *coli* S17 cells to *Rba. sphaeroides* WT (both for creating Δ*pufX* and Δ*pufY* mutants) and *Rba. sphaeroides* Δ*pufX* (using the Δ*pufY* plasmid; for creating Δ*pufXY mutants*) by conjugation. Selection of transconjugants was performed on M22 agar containing 30 μg·mL^–1^ kanamycin, and second recombinants were isolated on M22 medium containing 10% (w/v) sucrose. Successful generation of Δ*pufX*, Δ*pufY*, and Δ*pufXY* strains was confirmed using PCR using Q5 High-Fidelity DNA Polymerase (New England Biolabs, UK) and DNA sequencing (Eurofins).

*Rba. sphaeroides* wild-type (DSM 158) and the Δ*pufY* mutant were grown phototrophically under anoxic conditions in liquid M22+ medium^23^ supplemented with vitamins (0.08 M nicotinic acid, 0.01 M thiamine, 7.3 mM 4-aminobenzoic acid, and 0.4 mM d-biotin) and 0.1% casamino acids, at 29 °C in sealed glass bottles under a light intensity of 70 μmol photons s^–1^ m^–2^ provided by Bellight 70 W halogen bulbs. The nonphototrophic *Rba. sphaeroides* Δ*pufX* and Δ*pufXY* mutant was grown at 29 °C in the dark in the same medium under microoxic conditions in an orbital shaker set at 150 rpm.

The cells were harvested via centrifugation at 5,000*g* for 10 min at 4 °C, washed twice with Tris-HCl (pH 8) and resuspended in working buffer (20 mM HEPES-Na, pH 8.0). Cells were disrupted by passage through a French press three times at 16,000 psi. Cell debris was removed by centrifugation at 20,000*g* for 30 min. Membranes were collected by centrifuging the resulting supernatant at 125,000*g* for 90 min and were solubilized by the addition of DDM (*n*-dodecyl β-d-maltoside) to a final concentration of 3% (w/v) for 15 min in the dark at 4 °C with gentle stirring. After the insolubilized material was removed by centrifugation at 21,000*g* for 30 min, the clarified supernatant containing solubilized photosynthetic complexes was applied onto the 10–25% (w/v) continuous sucrose gradients made with working buffer containing 0.01% (w/v) DDM. Gradients were centrifuged at 230,000*g* for 19 h. For the WT dimers, the RC-LH1 complexes were collected and further purified by a Superose 6 gel filtration column (GE). For the WT monomers and mutants, the RC-LH1 complexes were collected and concentrated using Vivaspin 6 100,000 MWCO columns (Cytiva) for heavy complexes (dimers) and Vivaspin 6 50,000 MWCO columns for light complexes (monomers). Simultaneously, the buffer containing sucrose was exchanged with working buffer containing 0.01% (w/v) β-DDM.

TA spectroscopy was performed using a Harpia-TA spectrometer (Light Conversion). The probe and pump are generated using a PHAROS-SP-10W (Light Conversion) with a 1028 nm at 10 kHz and fwhm of approximately 170 fs. The pump beam is tuned to the desired wavelength using an OPA (Orpheus, Light Conversion) equipped with a second harmonic generation stage (Lyra, Light Conversion) and with a beam diameter of ca. 600 μm (1/e^2^ diameter) at the sample. The pump beam is chopped, resulting in an effective pump rate of 5 kHz. The white light probe is generated by focusing the 1028 nm beam onto a sapphire crystal and is focused to ca. 400 μm spot at the sample. The pump polarization was altered to ensure that the pump and probe beam interacts with the sample at the magic angle of 54.7° to eliminate the effect of anisotropy and rotational diffusion on the spectra.^24^ The spectra are recorded using a NMOS detector (S3901, Hamamatsu), following dispersion by a spectrograph (Kymera 193i, Andor). This configuration enables us to probe between 530 and 950 nm. A pump power of 50 μW (effective pumping rate of 5 kHz) was employed for all samples which reduced exciton–exciton annihilation (EEA) effects while maintaining a good signal-to-noise ratio required for data analysis.

The experiments were performed using a 2 mm path length quartz cuvette; the solution was not agitated for the duration of each experiment (1 h 20 min) and the signal remained stable. Samples were diluted to an OD of ∼0.1 in IMAC buffer containing 50 mM sodium ascorbate and 0.4 mM terbutryn. As reported for previous TA experiments on similar complexes the sodium ascobate acts as a sacrificial electron donor, whereas the terbutryn acts as Q_B_ inhibitor, ensuring Q_A_ remains reduced throughout the experiments.^25,26^ The pump wavelength was chosen to match the absorption maximum of the ^LH1^BChl(Q_y_) band observed in the steady-state UV/vis spectrum for each RC-LH1 supercomplex. Before each experiment, the sample was irradiated by the pump beam for 60 s to ensure that the RC Q_A_ was photochemically reduced to ensure that Q_A_ inactivation in the charge transfer relaxation process was probed within the RC.

The data were initially processed using CarpetView (Light Conversion) to account for chirp correction (performed using the response from a silicon wafer). Due to the experimental configuration, scattered pump light is detected. Owing to this, 15 nm on either side of the pump wavelength is cut from the spectra before performing subsequent analysis. The processed data were fitted with global lifetime analysis and following presmoothing in the time axis with a 5 nearest neighbor smoothing function, lifetime density analysis (LDA), using OPTIMUS.^27^ Only the 750–950 nm spectral region, which contains the dominating TA features, was included in the LDA fit; this considerably reduced computational resources required and prevented over smoothing owing to the introduction of low signal/noise data (as a result of the weak TA features observed <750 nm) see Supporting Information, Section 3. In all cases, the data were fitted employing 3 Gaussian coherent artifact signals.

## Results and Discussion

**Figure 2:**
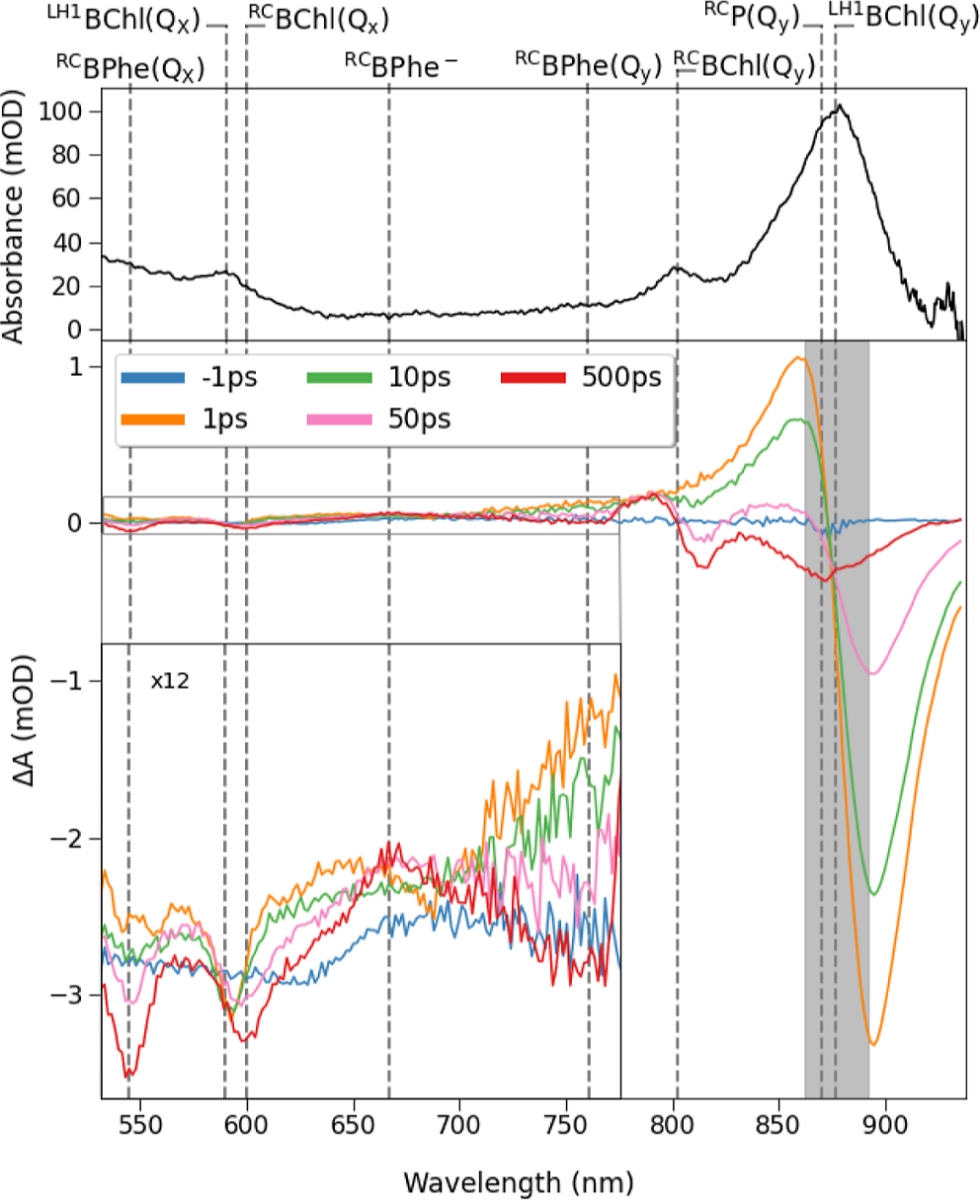
Spectral analysis of the WT RC-LH1 monomer. Top, UV/vis spectrum. Bottom, TA spectra at selected times, obtained for the WT RC-LH1 monomer at a pump wavelength 877 nm, coinciding with the of peak absorbance of the ^LH1^BChl(Q_y_) band. The 862–892 nm spectral region highlighted by the gray box is excluded from subsequent analysis owing to detection of scattered pump light. Bottom (Inset). TA spectra over the 525–775 nm spectral region magnified ×12.

The UV/vis and TA spectra recorded for the WT monomer are shown in Figure 2; those obtained for the WT dimer, Δ*pufY* monomer and dimer, and Δ*pufX* and Δ*pufXY* monomers are shown in Figures S3–S7 and show very similar features. The TA spectral features can be readily assigned based on those observed in the ground-state UV/vis spectra and those reported in TA spectra of other RC-LH1 complexes or isolated RCs.^17,25,28,29^ Briefly, at early times (1 and 10 ps; Figure 2, lower panel), all features are assigned to the LH1 chromophores. A feature with a derivative line shape at ∼877 nm is assigned to ^LH1^BChl(Q_y_). The negative band at ∼895 nm is attributed to the overlapping ground-state bleach (GSB) of ^LH1^BChl(Q_y_) and stimulated emission (SE) of ^LH1^BChl(Q_y_)*, while the positive feature, with a peak at ∼860 nm, is assigned to the photoinduced absorption (PIA) of ^LH1^BChl(Q_y_)*.^25^ A broad PIA was observed at wavelengths <750 nm, onto which a narrow, negative going feature at 590 nm is superimposed, assigned to the GSB of ^LH1^BChl(Q_*x*_).^29,30^

At later times (50 and 500 ps, Figure 2, lower panel), spectral features corresponding to RC chromophores appeared contemporaneously as the LH1 bands decayed, indicating that LH1 → RC EET has occurred. A derivative line shape feature centered at ∼800 nm was observed, coinciding with the absorption maximum of the ^RC^BChl(Q_y_) band in the ground-state UV/vis spectrum. Although ^RC^BChl chromophores are involved in the initial charge separation which occurs following LH1 → RC EET, ^RC^BChl is expected to remain reduced for <1 ps.^31,32^ This contrasts with the observation of the distinct feature with a point of inflection at 800 nm at later times (500 ps spectrum; Figure 2, lower panel). We conclude that this change can be attributed to the shift of the ^RC^BChl(Q_y_) band to shorter wavelengths than in the ground-state spectrum owing to a difference in the local environment as a result of charge transfer and subsequent localization within the RC.^33^ Superimposed on the broad PIA, the two negative bands at ∼545 and ∼760 nm were assigned to GSB of the Q_*x*_ and Q_y_^RC^BPhe bands, respectively, while the broad PIA at ∼665 nm was assigned to the formation of ^RC^BPhe^–^.^13,34^ The PIA at ∼860 nm observed at early times (1 and 10 ps, Figure 2) became a negative feature at later times, as observed in the 500 ps spectrum (Figure 2), and was assigned to GSB of ^RC^P(Q_y_).^13^ Finally, the negatively going band at ∼600 nm, which partially overlaps with the GSB band of ^LH1^BChl(Q_*x*_), was assigned to GSB of ^RC^BChl(Q_*x*_).^17^

We employed LDA and generated three-dimensional lifetime density maps, *x*(τ,λ), to examine the kinetics observed within the TA spectra of the RC-LH1 complexes (Figure S8). LDA enables us to monitor dispersive kinetics in a way which is not possible with global lifetime analysis (see Supporting Information discussion Section 7).^27,35,36^ However, comparing lifetime density maps is complicated owing to the difficulty in accurately representing the pre-exponential factor magnitude with contour/color maps.^37^ Hence, we reduced the three-dimensional lifetime density map to two-dimensional kinetic traces by integrating the modulus of the pre-exponential factor between 750 and 950 nm for each lifetime. This generated what we term a “lifetime density kinetic trace”, LDKT.

**Figure 3:**
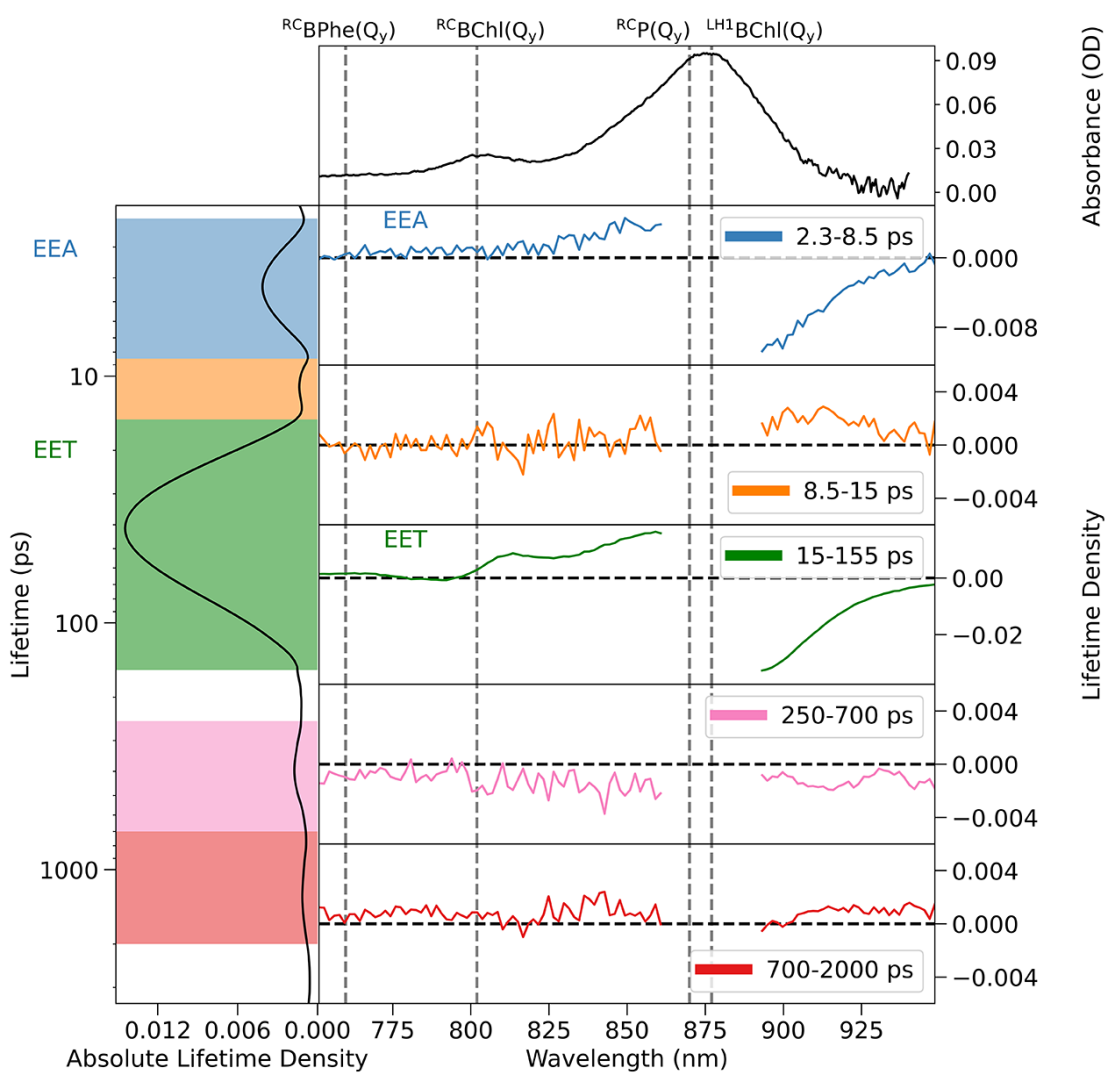
Lifetime density kinetic trace, LDKT, (left panel), UV/vis spectrum (top right), and lifetime averaged difference spectra (right panels 2–6) associated with the peaks observed in the LDKT, obtained for the WT RC-LH1 monomer. The lifetime averaged difference spectra panels show the wavelength-dependent average pre-exponential factor of lifetimes within the shaded area of same color.

By calculating the wavelength-dependent average pre-exponential factor of lifetimes associated with each band observed in the LDKT, we can plot the spectral change of each kinetic process in two dimensions, which we term “lifetime averaged difference spectra” (LADS). Figure 3 shows the LDKT and LADS obtained from LDA of a typical TA spectrum of the WT monomer RC-LH1 supercomplex. LADS indicates the change that occurs in the TA spectra throughout the distribution of lifetimes included within the average. Owing to this, a positive feature in the LADS indicates the decay of a positive TA band or the growth of a negative TA band, while a negative feature indicates the decay of a negative TA band or the growth of a positive TA band.

For each RC-LH1 complex studied, a dominant process with a peak at ∼40–60 ps, has an associated LADS which describes a loss of LH1 TA bands [loss of ^LH1^BChl(Q_y_) GSB and ^LH1^BChl(Q_y_)* SE signal at wavelengths longer than ∼890 nm and ^LH1^BChl(Q_y_)* PIA at ∼860 nm] contemporaneously with the growth of RC features [^RC^BChl(Q_y_) derivative line shape band at ∼800 nm and ^RC^BPhe(Q_y_) GSB at ∼760 nm], indicative of LH1 → RC EET. The LADS associated with the process at ∼4 ps show the decay of LH1 chromophore TA bands, consistent with EEA, as reported on similar time scales in the TA spectra of other RC-LH1 complexes,^13,25^ and ^LH1^BChl(Q_y_)* → ^LH1^BChl(Q_y_) relaxation. It is important to avoid excessive analysis of the weaker features observed within the LDKT, which may arise from residual noise in the analysis as well as the blurring of kinetic processes within the broad baseline. The LADS associated with other highlighted peaks in the LDKT are very weak, which did not allow us to assign these peaks to specific photophysical processes.

**Figure 4:**
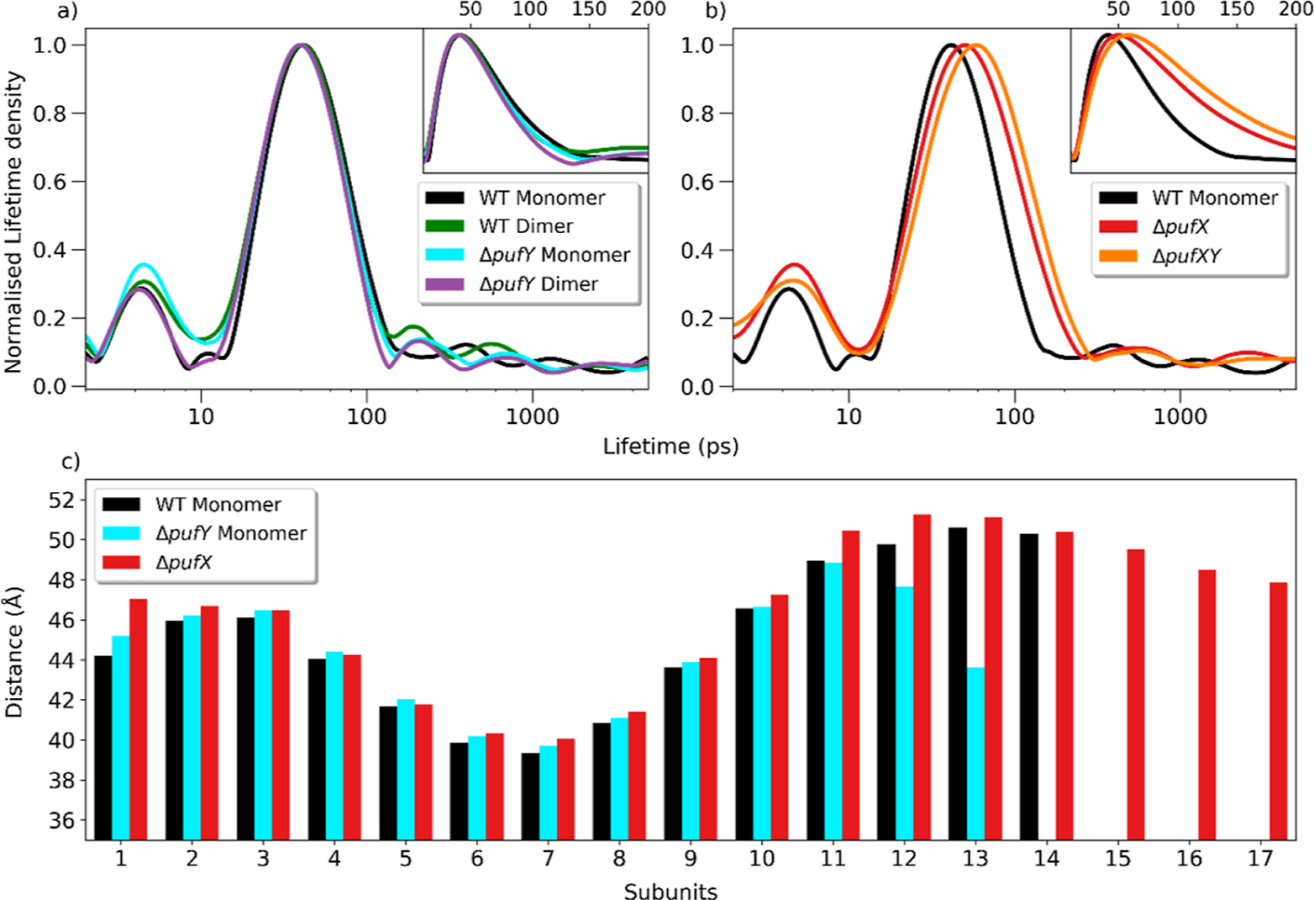
Comparison of the lifetime density kinetic traces and distance between LH1 and RC special pair Bchl *a* of the RC-LH1 complexes studied. (a) Comparison of the lifetime density kinetic traces WT monomer, WT dimer, Δ*pufY* monomer, and Δ*pufY* dimer. (b) Comparison of the lifetime density kinetic traces WT, Δ*pufX* and Δ*pufXY monomers*. (c) Average distances (measured between Mg ions) between each LH1 BChl *a* to the closest BChl *a* of the special pair for each of the monomeric RC-LH1 supercomplexes. The measurement for each BChl in the LH1 subunit is shown in Figure S23.

The LDKT and associated LADS obtained for the RC-LH1 supercomplex of the WT monomer are broadly representative of those obtained for the other supercomplexes studied, as shown in Figures S10–S14. LDKTs are shown in Figure 4a obtained from typical TA spectra of the WT and Δ*pufY* RC-LH1 monomers and dimers, which are dominated by LH1 → RC EET, with peak lifetimes, τ_EET_, of ∼40 ps. The τ_EET_ of the Δ*pufXY* RC-LH1 is longer than that of the Δ*pufX* RC-LH1 supercomplex, which is longer than that of the WT RC-LH1 monomer (Figure 4b). The average peak lifetime τ_EET_ and fwhm from the analysis of TA spectra for all RC-LH1 supercomplexes is summarized in Table S1.

The distribution of lifetimes associated with each kinetic process observed in the LDKT can provide important insight,^38^ with the τ_EET_ band notably broader for the Δ*pufX* and Δ*pufXY* RC-LH1 supercomplexes compared to that of the monomeric WT RC-LH1 supercomplex (Figure 4b). It is important to note that lifetime distributions obtained from LDA are particularly sensitive to noise within the data set and the regularization applied within the fit.^27,37^ We performed Global Lifetime Analysis of the TA spectra, in which the EET process is well described by a single lifetime compartment for the monomeric and dimeric WT and Δ*pufY* RC-LH1, (Figures S15 and S17), whereas two EET lifetime compartments are required to adequately describe the TA data obtained from the Δ*pufX* and Δ*pufXY* RC-LH1 monomers (Figures S16 and S17). Such a behavior is consistent with the increased kinetics distribution of LH1 → RC EET for the Δ*pufX* and Δ*pufXY* RC-LH1 supercomplexes,^36^ as identified by LDKT.

Although the structure of the Δ*pufXY* monomer remains unreported, an analysis of the structures of the other RC-LH1 supercomplexes has been previously reported.^5^ We performed additional detailed analysis of these reported structures (Figures 4c and S21–26 and Table S2). This identified that in addition to the loss of one LH1 subunit, the BChl chromophores of three LH1 subunits in the Δ*pufY* monomer (LH1-11, LH1-12 and LH1-13) shift inward toward ^RC^P by between 3.3 and 7.6 Å compared to those in the WT monomer, whereas there is no significant difference for other ^LH1^BChls (Figure 4c). This might be expected to result in a decrease in the EET lifetime for Δ*pufY* monomer.^4,39–43^ However, the TA analysis reveals that τ_EET_ and distributions remain remarkably similar (Figure 4a), and tentatively, may even display a small increase in lifetime (Table S1). The shift toward the RC is even larger for several LH1 chromophores of the Δ*pufY* dimer, with a shift of over 10 Å observed in LH1-13 and LH1-14 subunits (Figure 4c). Despite this, the EET lifetime and distribution obtained are indistinguishable from that of the WT dimer (Figure 4a) despite two very differing structures being obtained for the Δ*pufY* dimer (Figure S1). Based on these observations, we found that there is no clear correlation between the changing number of ^LH1^BChl chromophores and ^LH1^BChl-^RC^P distances that occur upon the removal of the PufY TM protein determined from cryo-EM (88 K) on the EET lifetime or distribution obtained at 294 K, consistent with the finding of *Rhodopseudomonas palustris* RC_3_-LH1_14_-W and RC_3_-LH1_16_ complexes.^25^ Even though there are differences in τ_EET_ between the WT and Δ*pufX* supercomplexes (Figure 4b), only very small increases in ^LH1^BChl-^RC^P distances occur upon the removal of the PufX TM protein (Figure 4c).

The average inter- and intramolecular distances between ^LH1^BChl chromophores in the monomeric Δ*pufY* and Δ*pufX* RC-LH1 are remarkably similar but differ from those in the WT supercomplex (Figure S21 and Table S2). Such a difference may influence the degree of delocalization/localization of both initially formed and thermalized excitonic states between the RC-LH1 supercomplexes,^5,44^ which in turn could account for the observed differences in EET kinetics. Notably, the ^LH1^BChl(Q_y_) peak absorptions of the monomeric WT, Δ*pufY*, and Δ*pufXY* RC-LH1 are remarkably similar (Figure S18), whereas a notable spectral change occurred at the wavelength of 480–550 nm owing to the conversion of the carotenoid spheroidene to spheroidenone in the Δ*pufX* and Δ*pufXY* strains grown under the microoxic culture conditions, consistent with previous findings.^5,29,45^ Although a small shift (∼1 nm) to shorter wavelengths was observed for the ^LH1^BChl(Q_y_) absorption maximum of the Δ*pufX* RC-LH1 monomer, this brings the ^LH1^BChl(Q_y_) peak closer in wavelength to the ^RC^P(Q_y_) absorption, which may be expected to decrease τ_EET_.^4,41,46^ The 900 nm kinetic traces for all monomeric RC-LH1 complexes are shown in Figure S20; the increased decay observed between 1 and 10 ps for the Δ*pufX* and Δ*pufXY* complexes is a result of the greater EEA observed for these samples (Figures 4b, S6, and S7), which arise owing to the increased probability of absorbing a second photon ascribed to the increased number of BChl *a* chromophores within LH1 of these supercomplexes. Although we cannot exclude the possibility that differing initial kinetic processes occur within our instrument response function (∼350 fs) for the RC-LH1 complexes studied, the positions and shapes of the TA spectra at 250 fs and 10 ps were remarkably similar (within a spectral resolution of ∼3.5 nm and precision of ∼1.7 nm) for all monomeric supercomplexes (Figure S19). These results suggest that the initially formed excitons and subsequent relaxation processes are comparable across all RC-LH1 variants and are likely not the cause of the observed differences in EET kinetics.

The EET lifetime within RC-LH1 supercomplexes has been suggested to be dependent on the orientation of the RC with the LH1 ring.^47^ Only a relatively lower-resolution cryo-EM structure (>4.2 Å) was obtained for the Δ*pufX* RC-LH1, in which, notably, the RC exhibited weak density, indicating the role of PufX in constraining the RC association and orientation resulting in a less defined RC orientation within the LH1 ring in Δ*pufX* RC-LH1.^5^ We propose that the increased EET lifetime and distribution observed in our TA results obtained for the Δ*pufX* RC-LH1 complex are due to this less defined orientation of the RC within the LH1 ring of this complex. By contrast, the τ_EET_ and distribution remain very similar upon removal of only PufY, suggesting that PufY has a minor role in constraining the RC orientation within LH1. Removal of both PufX and PufY resulting in a further increase in the EET lifetime and distribution (Figure 4a,b and Table S1) further confirms that the RC is considerably less constrained within the LH1 ring in the Δ*pufXY* RC-LH1 complex. Despite the longer EET lifetime for both Δ*pufX* and Δ*pufXY* RC-LH1 complexes, it is still able to outcompete LH1* radiative relaxation which has been reported to take longer than 500 ps;^48–51^ hence, we conclude that the observed differences in the initial LH1 → RC energy transfer is not responsible for the inability of the Δ*pufX* and Δ*pufXY* strains to grow photosynthetically.

## Conclusions

In summary, our results show that the structural changes caused by the absence of PufX resulted in a marked increase in the LH1 → RC EET lifetime and distribution of the *Rba. sphaeroides* RC-LH1; the absence of both PufX and PufY further increases the EET lifetime and distribution. Additionally, despite some ^LH1^BChl chromophores moving closer to the RC upon removal of PufY alone, we observed little effect on the EET lifetime and distribution. Our findings indicate that both integral components play roles in EET of the RC-LH1 supercomplex. It also suggests that PufX, and, to a lesser extent, PufY, could represent natural engineering targets for fine-tuning EET of the photosynthetic RC-LH1 core supercomplex.

## Supporting Information Available

Additional structural analysis; results of global lifetime analysis; and additional lifetime density analysis results of WT dimer, Δ*pufY* monomer and dimer, and Δ*pufX* and Δ*pufXY* monomers (PDF)
