# Supplementary material for: Unravelling the Roles of Integral Polypeptides in Excitation Energy Transfer of Photosynthetic RC-LH1 Supercomplexes
Source: J Phys Chem B. 2023 Aug 9;127(33):7283–90. doi: 10.1021/acs.jpcb.3c04466 (PMC10461223; doi:10.1021/acs.jpcb.3c04466)
Supplement: Supplementary file 1 — jp3c04466_si_001.pdf [file jp3c04466_si_001.pdf]

Electronic supplementary information for:

Unravelling the Roles of Integral Polypeptides in Excitation Energy Transfer of Photosynthetic RC-LH1 Supercomplexes

Owen Thwaites,[a,b,†] Bern M. Christianson,[c,†] Alexander J. Cowan,[a,d] Frank Jäckel,[a,b,*] Lu-Ning Liu,[c, e,*] and Adrian M. Gardner,[a, d,f,*]
[a]Stephenson Institute of Renewable Energy, University of Liverpool, Liverpool, L69 7ZF, UK
[b]Department of Physics, University of Liverpool, Liverpool, L69 7ZE, UK
[c]Institute of Systems, Molecular and Integrative Biology, University of Liverpool, Liverpool, L69 7ZB, UK
[d]Department of Chemistry, University of Liverpool, Liverpool, L69 7ZD, UK
[e]College of Marine Life Sciences, and Frontiers Science Center for Deep Ocean Multispheres and Earth System, Ocean University of China, Qingdao 266003, China
[f]Early Career Laser Laboratory, University of Liverpool, Liverpool, L69 3BX, UK

[†]These authors contributed equally
*Authors to whom correspondence should be addressed: Frank.Jaeckel@liverpool.ac.uk; luning.liu@liverpool.ac.uk; Adrian.gardner@liverpool.ac.uk

Contents

1. cryo-EM Structure S2
2. TA/UV-Vis Spectra S4
3. Analysis of the Transient Absorption Data S9
4. Lifetime Density Maps S10
5. L-Curve S11
6. LADS S12
7. Analysis of kinetic processes with broad distributions S18
8. LH1 peak shift S22
9. 900 nm Kinetic Trace S23
10. Structural Analysis S24

1. cryo-EM Structure

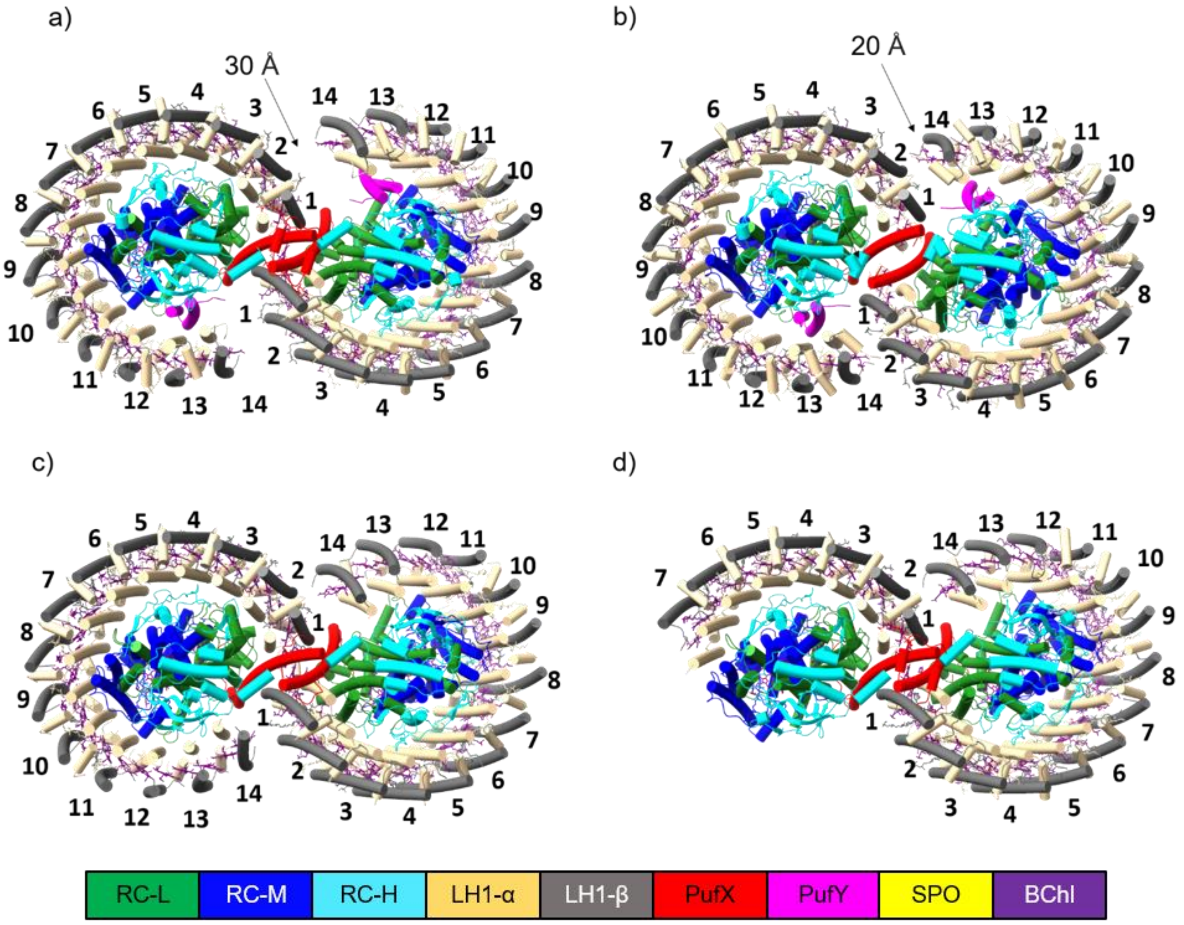

Figure S1. Cryo-EM structures of the RC-LH1 dimeric supercomplexes from *Rba. sphaeroides* viewed from the cytoplasmic side a) WT dimer type 1 (PDB ID: 7VOR), b) WT dimer type 2 (PDB ID: 7VOT), c) Δ*pufY* dimer type 1 (PDB ID: 7VA9), d) Δ*pufY* dimer type 2 (PDB ID: 7VB9)

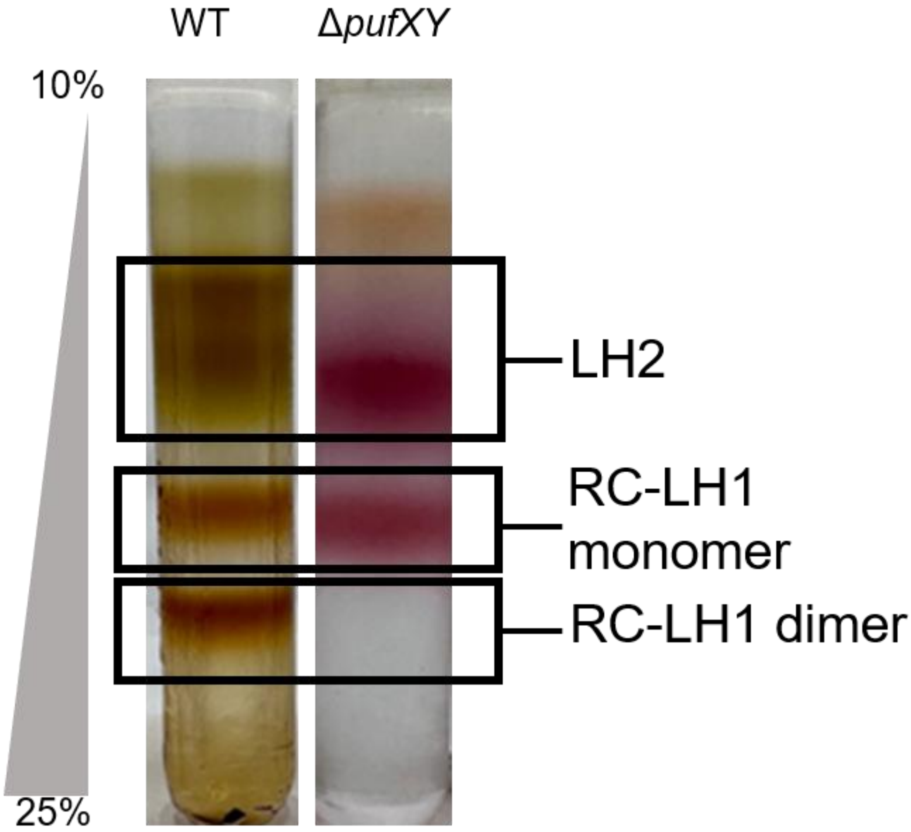

Figure S2. Separation of photosynthetic membrane complexes from *Rba sphaeroides* WT and Δ*pufXY*, using a 10-25 % continuous sucrose gradient. The RC-LH1 core complexes present as exclusively monomers in Δ*pufXY*. The separation of membrane proteins of the other species were identical to those reported previously. [1]

2. TA/UV-Vis Spectra

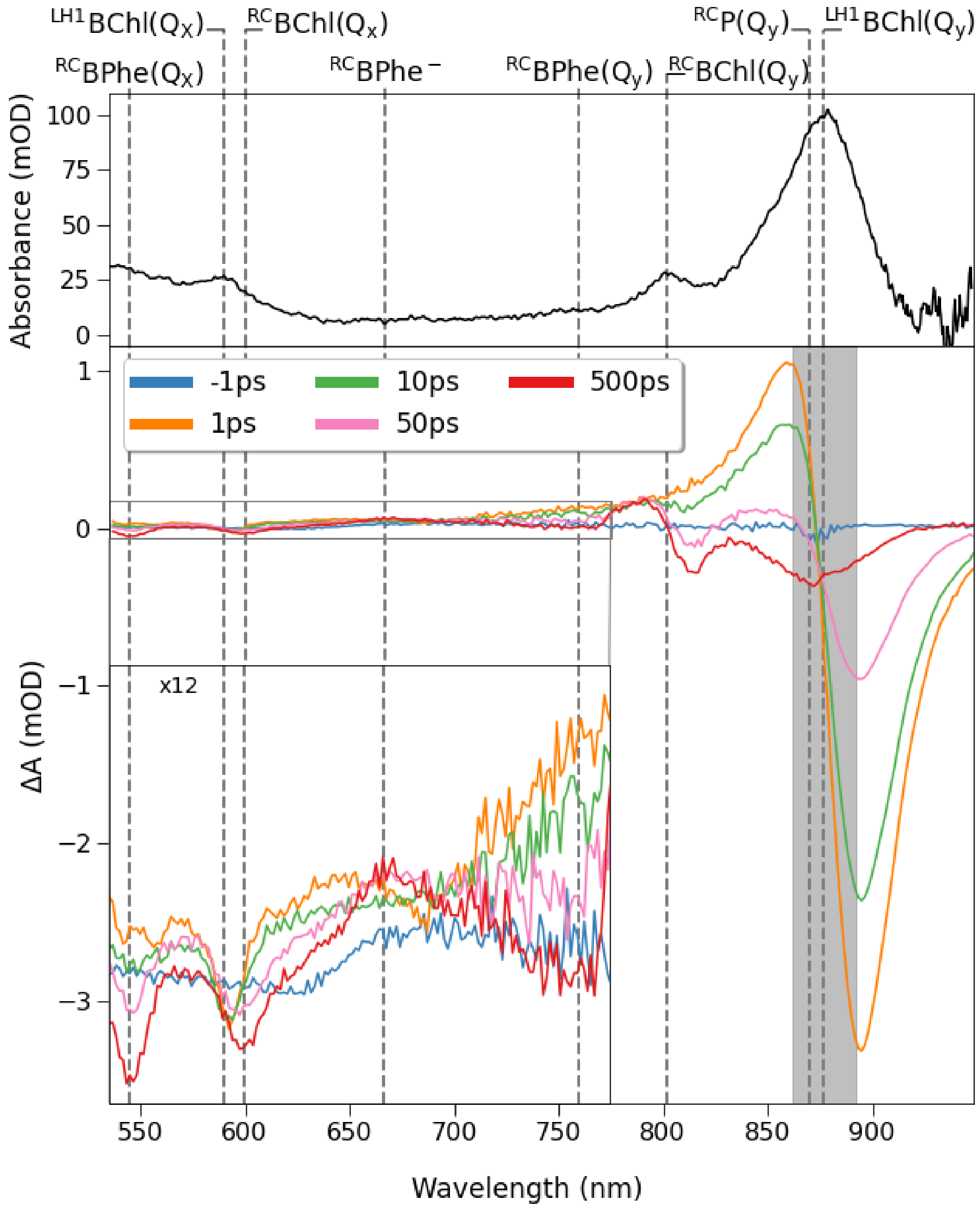

Figure S3. Top. UV/Vis spectrum of dimeric WT RC-LH1 supercomplex. Bottom. TA spectra at selected times, obtained for the dimeric WT RC-LH1 supercomplex at a pump wavelength 877 nm, coinciding with the of peak absorbance of the $^{LH1}BChl(Q_y)$ band. The 862-892 nm spectral region highlighted by the grey box is excluded from subsiquent analysis owing to detection of scattered pump light. Bottom (Inset) TA spectra over the 525-775 nm spectral region magnifided x 12.

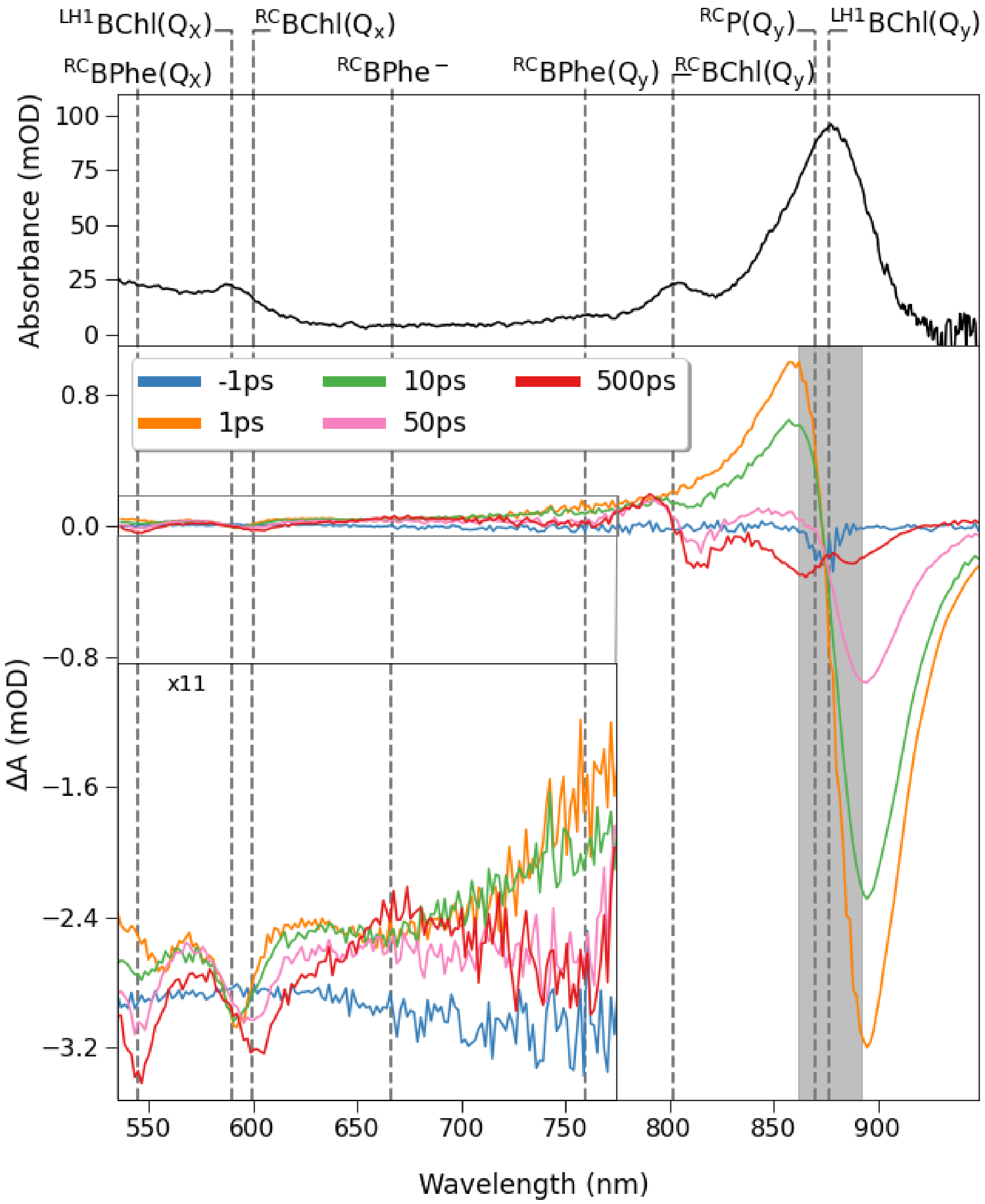

Figure S4. Top, UV/Vis spectrum of the monomeric *ΔpufY* RC-LH1 supercomplex. Bottom, TA spectra at selected times, obtained for the monomeric *ΔpufY* RC-LH1 supercomplex at a pump wavelength 877 nm, coinciding with the of peak absorbance of the ^{LH1}BChl(Q_y) band. The 860-890 nm spectral region highlighted by the grey box is excluded from subsiquent analysis owing to detection of scattered pump light. Bottom (Inset), TA spectra over the 525-775 nm spectral region magnifided x 11.

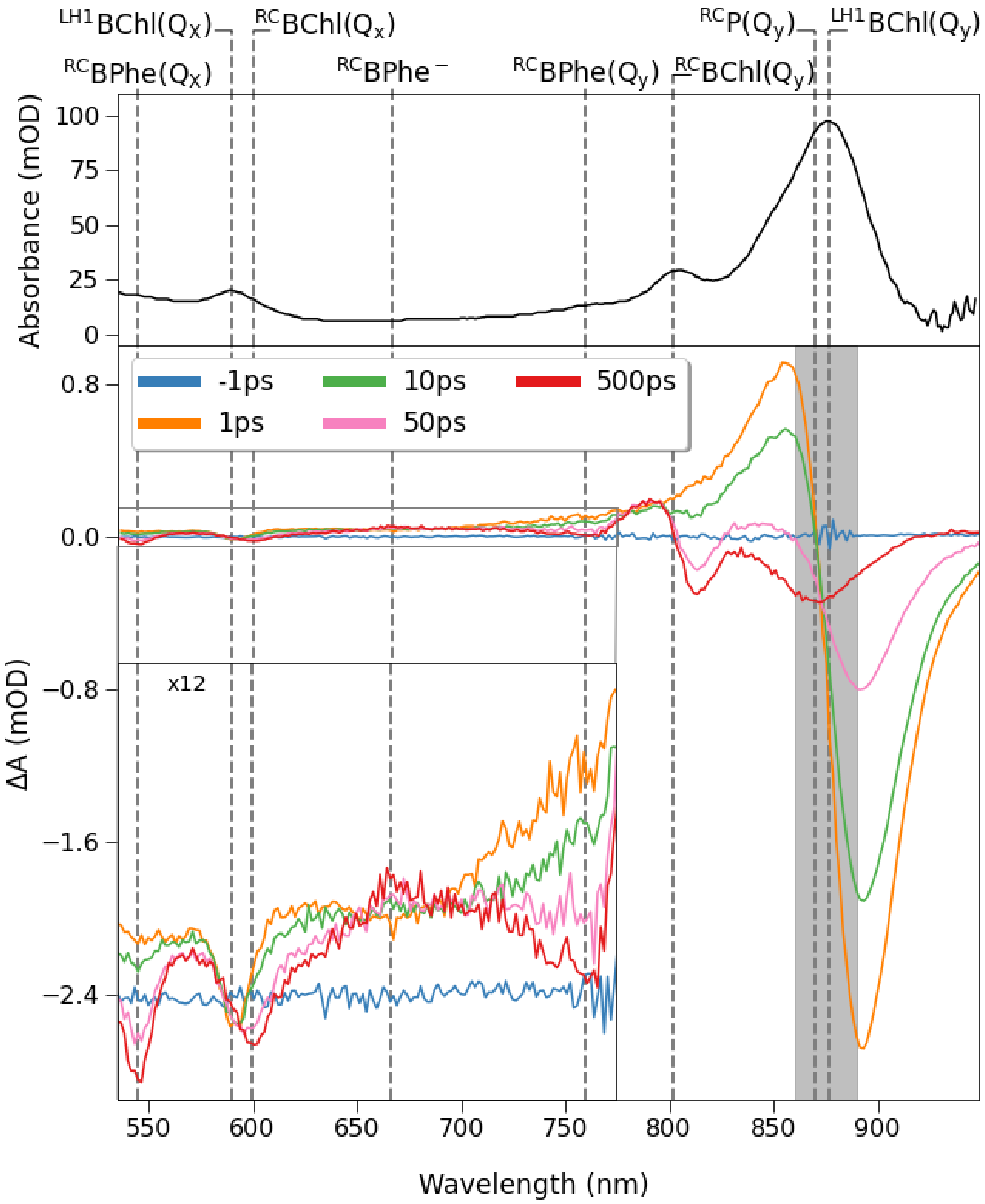

Figure S5. Top, UV/Vis spectrum of the dimeric *ΔpufY* RC-LH1 supercomplex. Bottom, TA spectra at selected times, obtained for the dimeric *ΔpufY* RC-LH1 supercomplex at a pump wavelength 877 nm, coinciding with the of peak absorbance ^{LH1}BChl(Q_y) band. The 862-892 nm spectral region highlighted by the grey box is excluded from subsiquent analysis owing to detection of scattered pump light. Bottom (Inset) TA spectra over the 525-775 nm spectral region magnifided x 12.

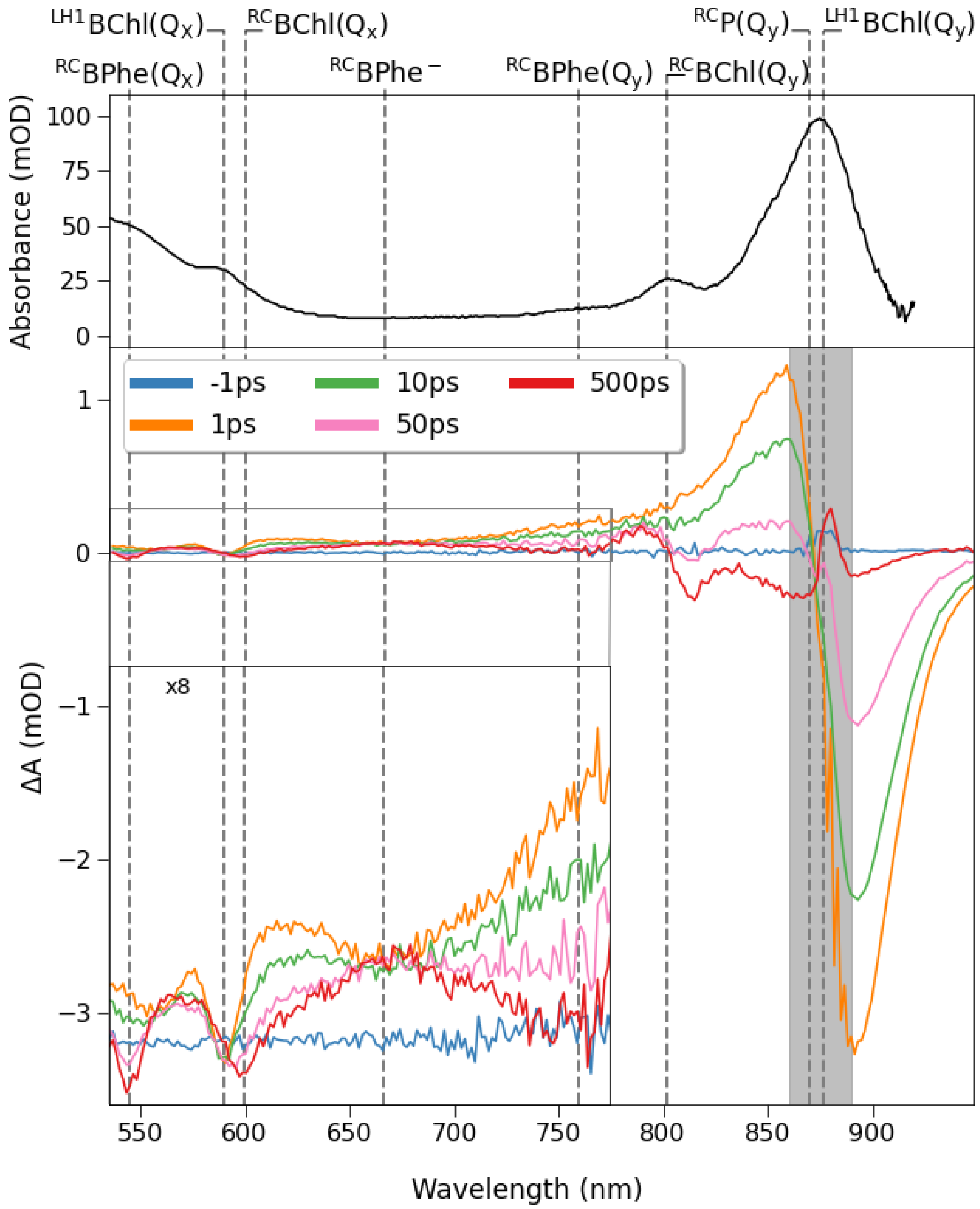

Figure S6. Top, UV/Vis spectrum of the monomeric *ΔpufX* RC-LH1 supercomplex. Bottom, TA spectra at selected times, obtained for the monomeric *ΔpufX* RC-LH1 supercomplex at a pump wavelength 877 nm, coinciding with the of peak absorbance ^{LH1}BChl(Q_y) band. The 862-892 nm spectral region highlighted by the grey box is excluded from subsiquent analysis owing to detection of scattered pump light. Bottom (Inset), TA spectra over the 525-775 nm spectral region magnifided x 9.

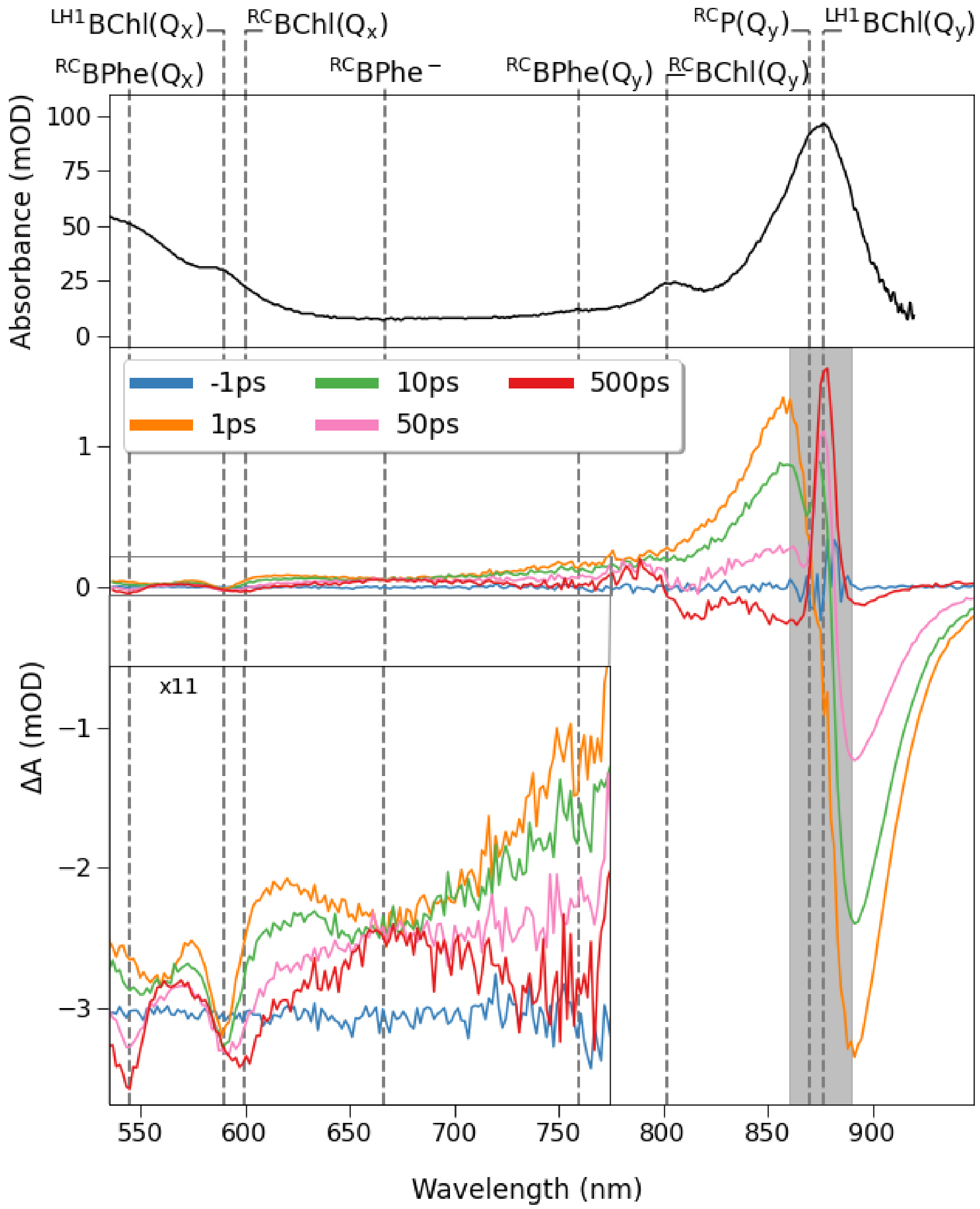

Figure S7. Top. UV/Vis spectrum of the monomeric *ΔpufXY* RC-LH1 supercomplex. Bottom, TA spectra at selected times, obtained for the monomeric *ΔpufXY* RC-LH1 supercomplex at a pump wavelength 877 nm, coinciding with the of peak absorbance ^{LH1}BChl(Q_y) band. The 862-892 nm spectral region highlighted by the grey box is excluded from subsiquent analysis owing to detection of scattered pump light. Bottom (Inset), TA spectra over the 525-775 nm spectral region magnifided x 12.

3. Analysis of the Transient Absorption Data

Several methods are commonly employed to provide kinetic insight into complex datasets generated from time-resolved spectroscopies. These have been reviewed recently.[2–6] Briefly, the simplest method is to fit kinetic traces at a single wavelength to a series of exponential decay/growth functions.[7] Alternatively, global fitting procedures, such as global lifetime analysis (GLA) offer a route to analyse the kinetics of all wavelengths simultaneously. GLA provides a route to visualise the complex time-resolved spectra by decomposing them into a small number (~2-5) of compartment populations. The lifetime of each compartment is fitted as a single exponential decay function, with the pre-exponential factor (x_j) allowed to vary with wavelength, Equation 1.[4] Conventionally, GLA results in decay-associated difference spectra (DADS) if the compartments are allowed to decay in parallel. The DADS are presented as plots of pre-exponential factor *vs*. wavelength, providing a representation of the change to the time-resolved spectra for each lifetime component, τ_j.[4]

$$\Delta A(t,\lambda) = \sum_{j=1}^{n} x_i(\tau_j,\lambda) e^{-\frac{t}{\tau_j}} \quad (1)$$

In this work we employ lifetime density analysis (LDA) to examine the RC–LH1 kinetics. LDA is based on the principle that the time-resolved data can be represented by a continuous distribution of single exponential functions, Equation 2, where $\emptyset(\tau)$ is the spectral distribution function.[5] To make Equation 2 readily solvable, the integral needs to be discretized into a quasi-continuous sum of *n* exponential functions (*n* = 500 in this work), becoming analogous to Equation 1. Although this discretization indicates that LDA and GLA are similar, and differ only by the value of *n*, an important distinction is that although both methods are effectively model independent, LDA requires no user input into the expected number of kinetic pathways and their associated lifetimes. [2,5]

$$\Delta A(\tau,\lambda) = \int_0^{\infty} \emptyset(\tau,\ \lambda)\, e^{-\frac{t}{\tau}} \qquad (2)$$

LDA results in a three-dimensional density map, $x(\tau,\lambda)$, termed a "lifetime density map", shown in Figure S8 for all six RC-LH1 complexes. Comparison of lifetime density maps is complicated owing to the difficulty in accurately representing the magnitude of the pre-exponential factor with contour/colour maps. Instead, we reduce the three-dimensional lifetime density map to a series of two-dimensional plots. Information on the kinetics is obtained through the integration of the modulus of the pre-exponential factor between 750 – 950 nm for each lifetime, (which displays the most intense TA spectra features), which we term lifetime density kinetic trace, LDKT. The wavelength dependent average pre-exponential factor of lifetimes associated with each band observed in the LDKT can be calculated, allowing the spectral change associated with each kinetic process to be plotted in two dimensions, which we denote as lifetime averaged difference spectra, LADS. LDKT and LADS are shown in Figure 3 obtained from LDA of a typical TA spectrum of the WT monomer RC-LH1 supercomplex and Figure S11 – S14 obtained from typical TA spectra of the other RC-LH1 complexes studied. LADS indicate the change that occurs in the TA spectra throughout the distribution of lifetimes included within the average. Owing to this, a positive feature observed within LADS indicates the decay of a positive TA band, or growth of a negative TA band. Similarly, a negative feature indicates the decay of a negative TA band, or the growth of a positive TA band.

4. Lifetime Density Maps

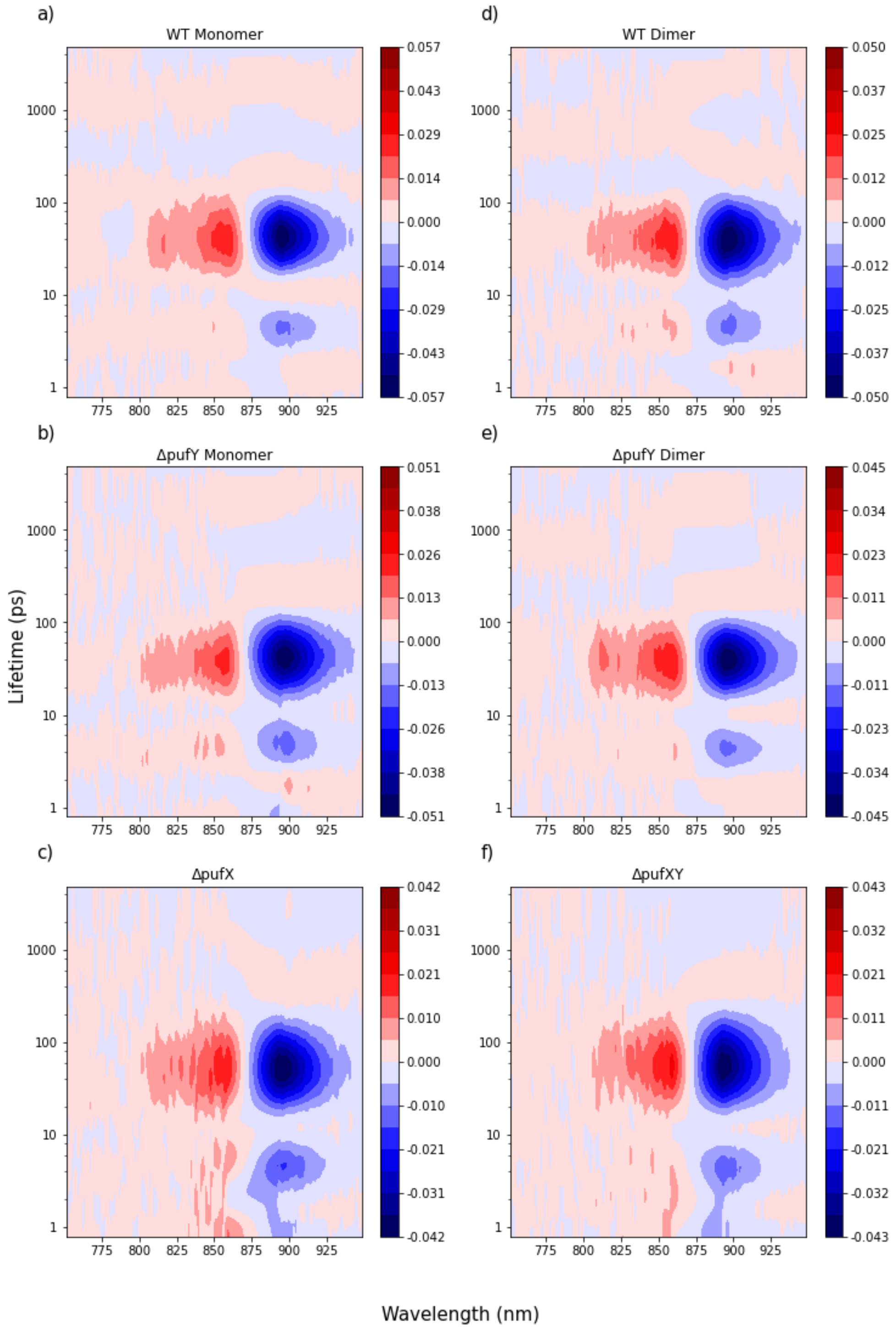

Figure S8. Lifetime density map generated from LDA of TA spectra for RC-LH1 complexes. a) WT monomer, b) *ΔpufY* monomer, c) *ΔpufX*, d) WT dimer, e) *ΔpufY* dimer, and f) *ΔpufXY*. With the colour bar showing the lifetime amplitude. The L-curves for each LDA fit are shown in figure S9. LDA fits performed over 750-950 nm region of TA spectra smoothed by 5 nearest neighbours over the whole spectral window apart from 15 nm on either side of the pump wavelength which is contaminated by pump scatter. The TA spectra are fitted with 500 lifetimes spread on a log scale between 0.03 ps to 20 ns. Lifetimes < 0.9 ps have been cut for clarity as at early timeframes LDMs are dominated by features assignable to remaining coherent artefact components.

5. L-Curve

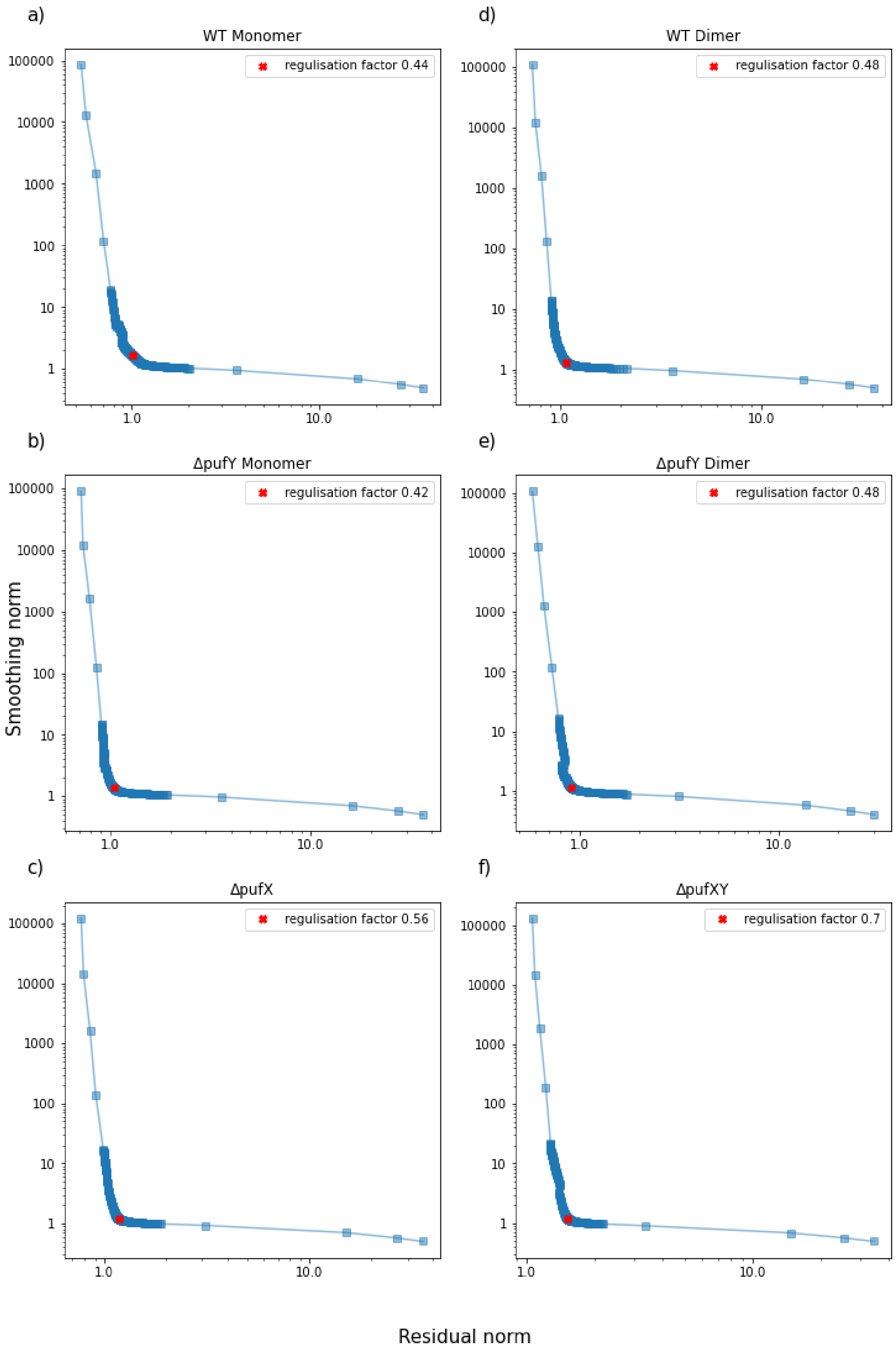

Figure S9. L-curves obtained from the LDA fitting process for RC-LH1 complexes. a) WT monomer, b) *ΔpufY* monomer, c) *ΔpufX*, d) WT dimer, e) *ΔpufY* dimer, and f) *ΔpufXY*. The point indicated by the red cross at the minima of the L-curve is the regulisation factor from which the lifetime density maps in Figure S8 are obtained.

6. LADS

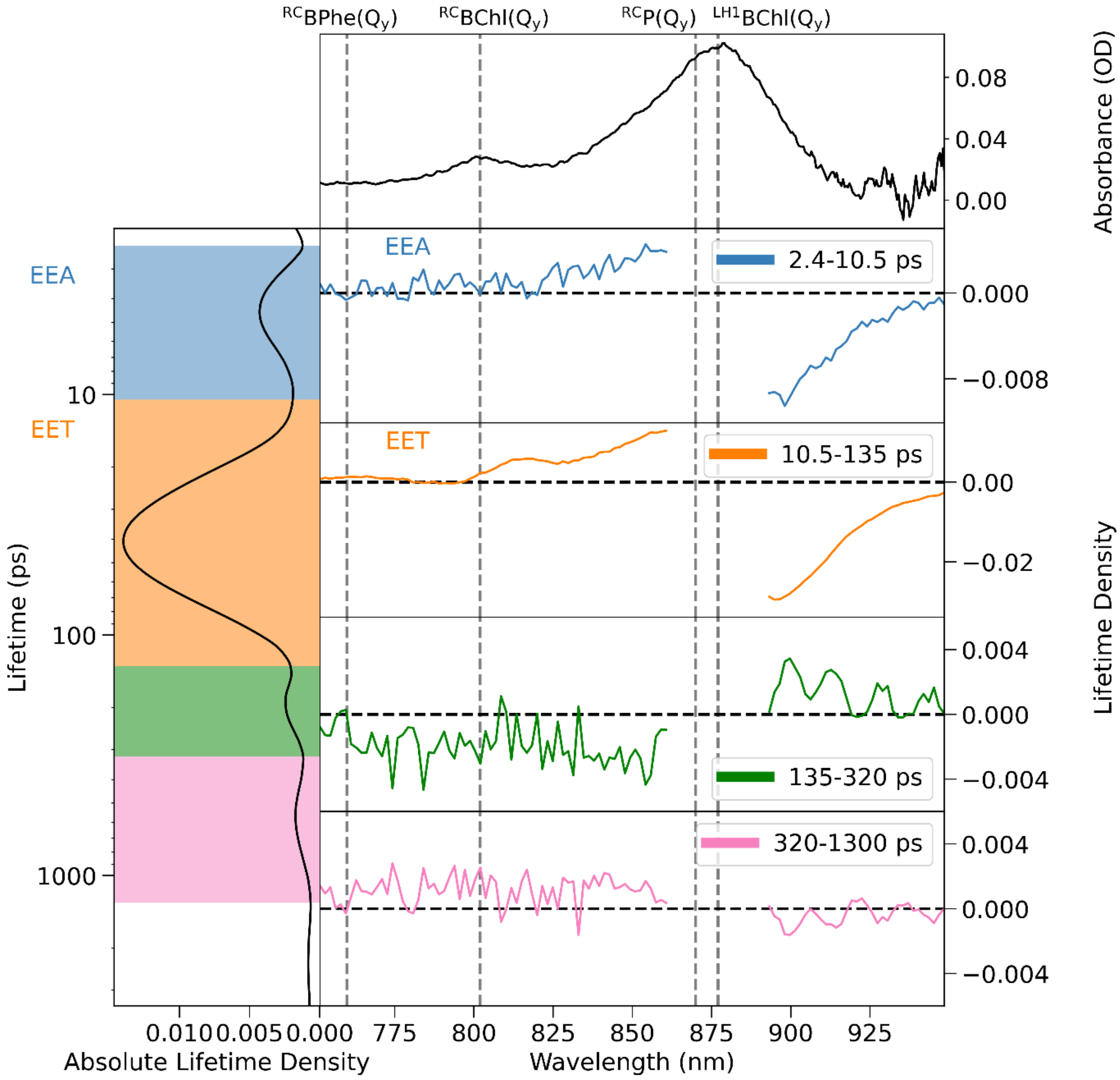

Figure S10. Combined lifetime density kinetic traces (left panel), UV-VIS (top right) and lifetime averaged difference spectra (right panel 2 to 5) of WT dimer. The lifetime averaged difference spectra panels show the wavelength dependent average pre-exponential factor of lifetimes within the shaded area of same colour.

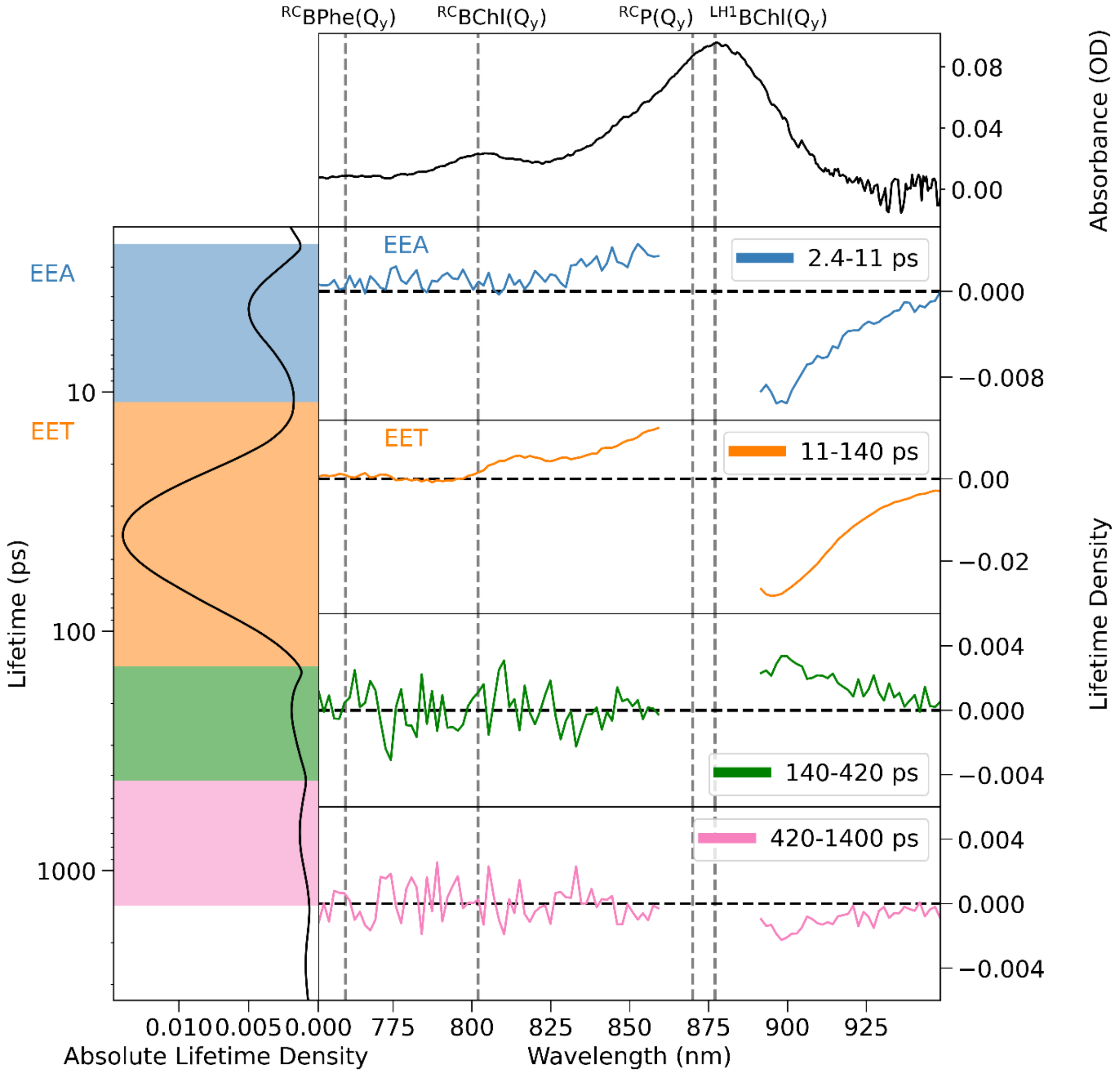

Figure S11. Combined lifetime density kinetic traces (left panel), UV-VIS (top right) and lifetime averaged difference spectra (right panel 2 to 5) of Δ*pufY* monomer. The lifetime averaged difference spectra panels show the wavelength dependent average pre-exponential factor of lifetimes within the shaded area of same colour.

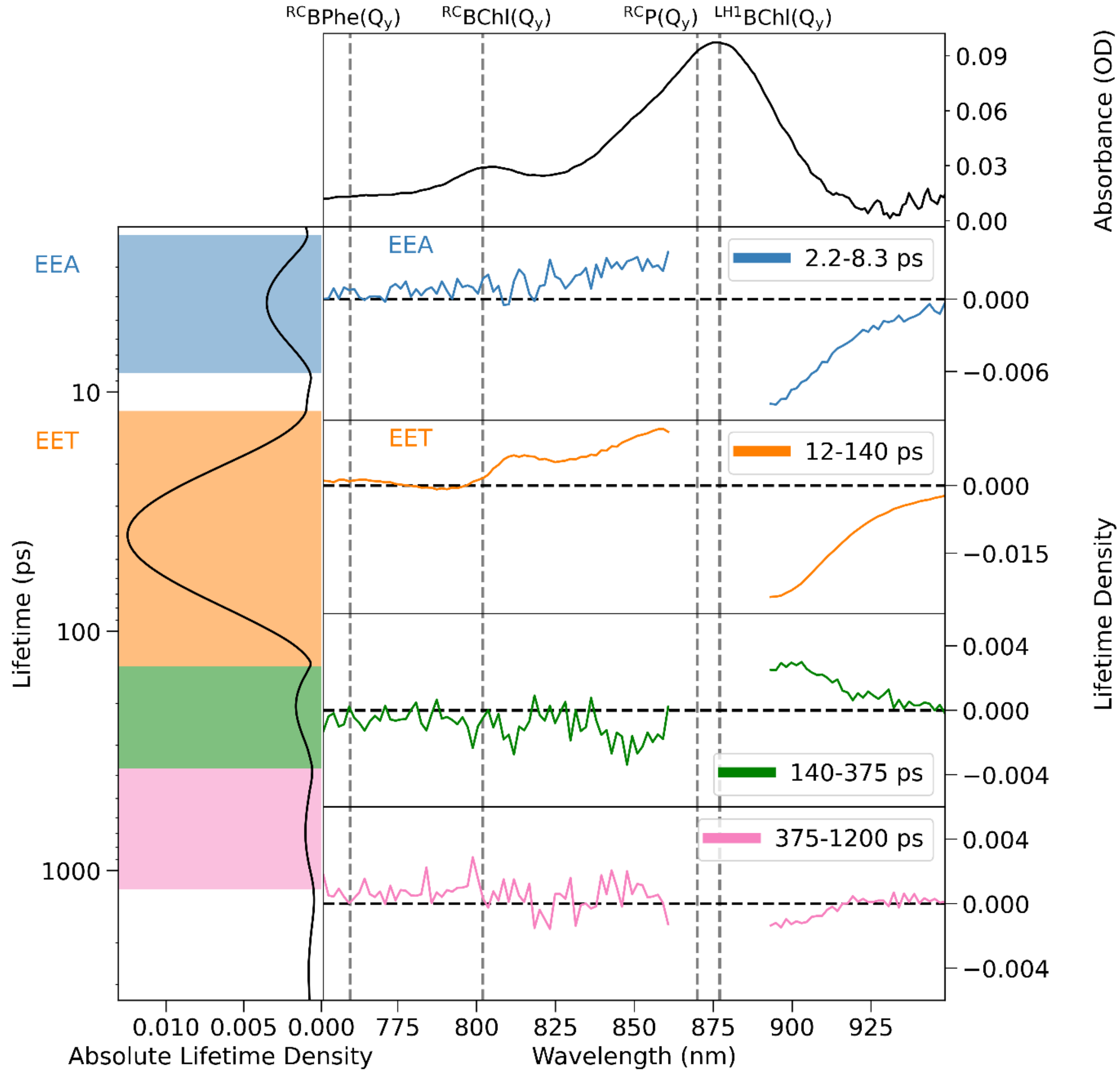

Figure S12. Combined lifetime density kinetic traces (left panel), UV-VIS (top right) and lifetime averaged difference spectra (right panel 2 to 5) of *ΔpufY* dimer. The lifetime averaged difference spectra panels show the wavelength dependent average pre-exponential factor of lifetimes within the shaded area of same colour.

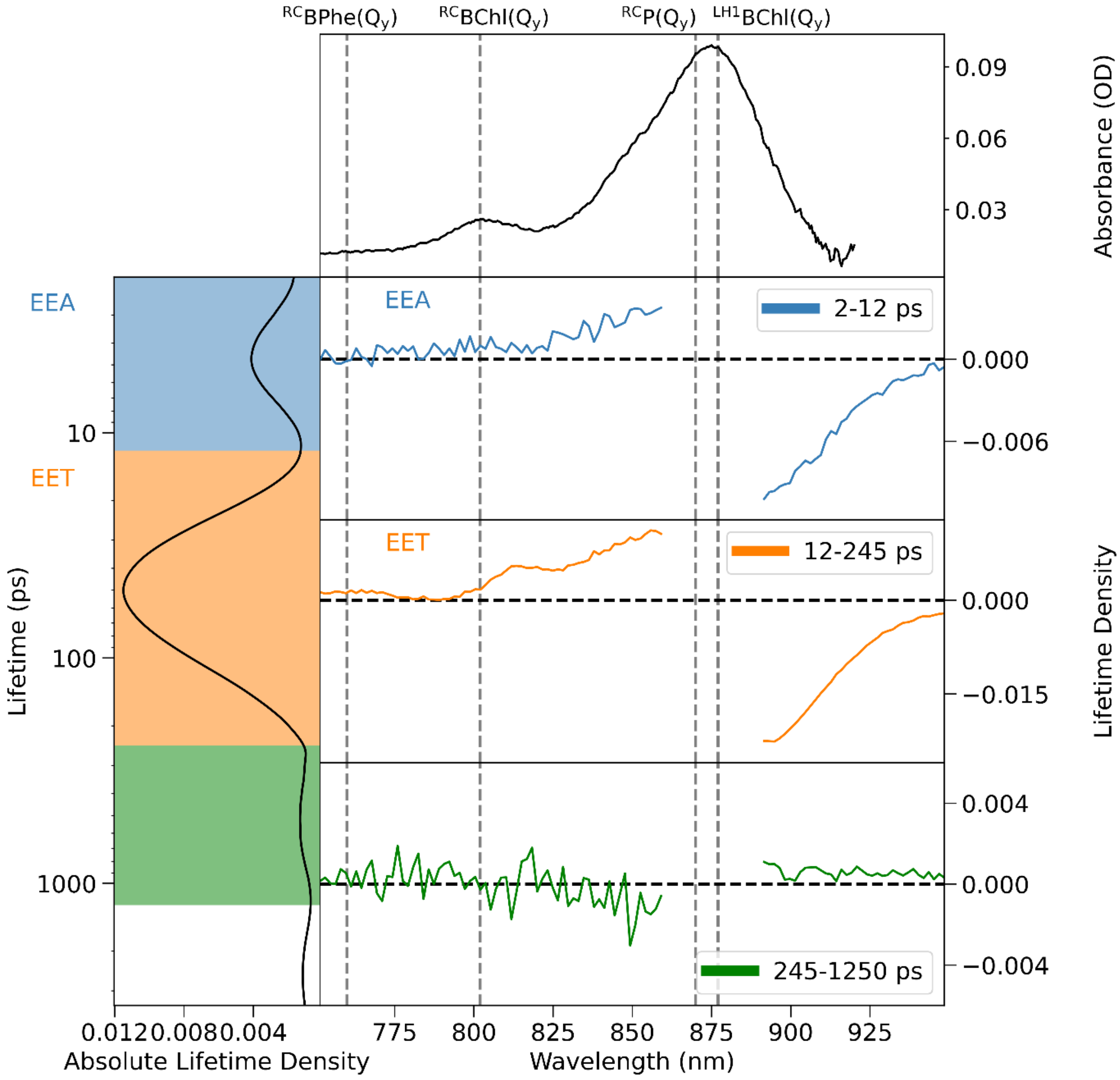

Figure S13. Combined lifetime density kinetic traces (left panel), UV-VIS (top right) and lifetime averaged difference spectra (right panel 2 to 4) of *ΔpufX* monomer. The lifetime averaged difference spectra panels show the wavelength dependent average pre-exponential factor of lifetimes within the shaded area of same colour.

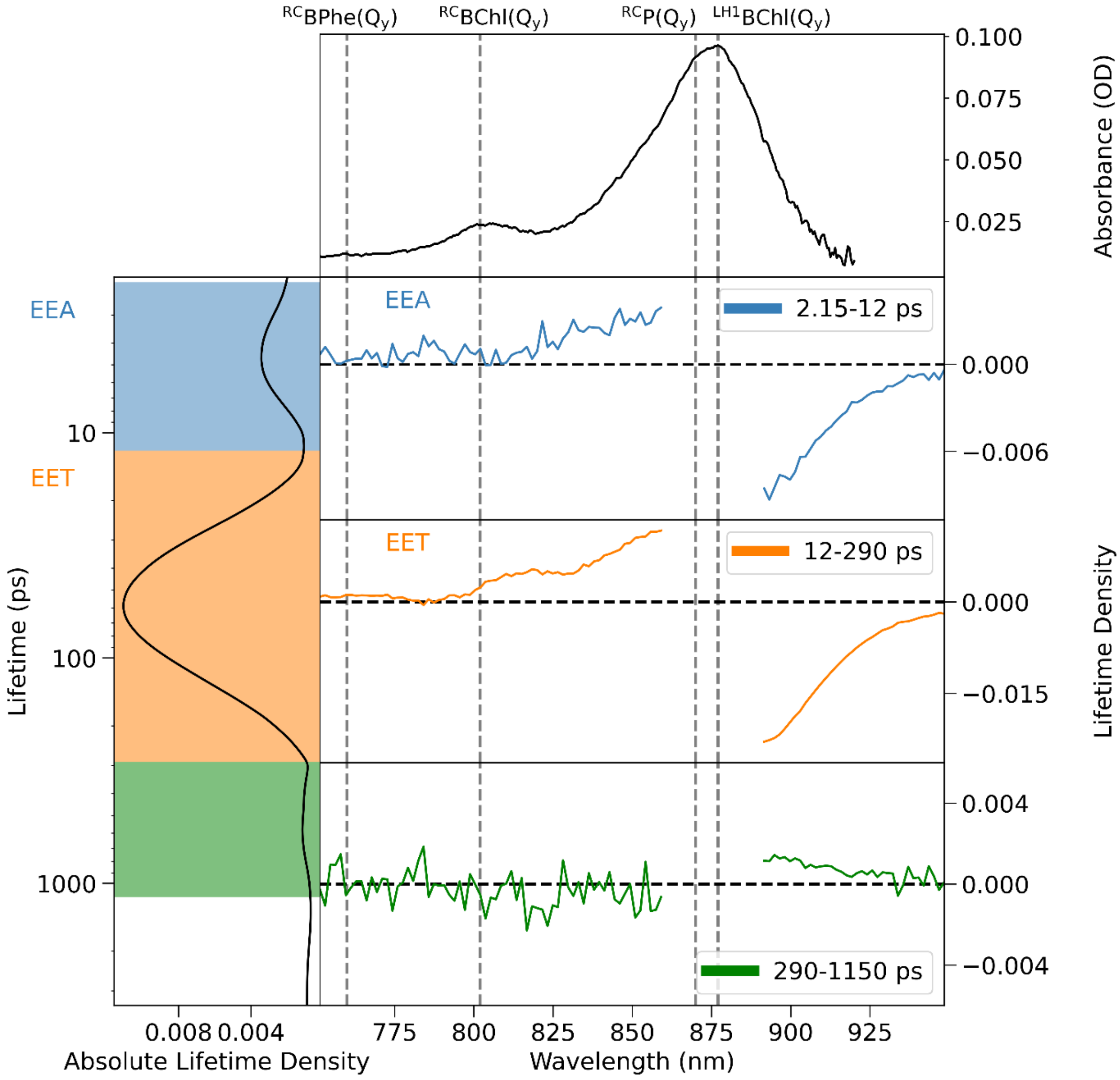

Figure S14. Combined lifetime density kinetic traces (left panel), UV-VIS (top right) and lifetime averaged difference spectra (right panel 2 to 4) of *ΔpufXY* monomer. The lifetime averaged difference spectra panels show the wavelength dependent average pre-exponential factor of lifetimes within the shaded area of same colour.

Table S1. Average peak lifetimes and FWHM, along with corresponding standard deviation in parentheses, for the EET process obtained from the lifetime density kinetic traces which result from the analysis of the TA spectra obtained from three samples of each RC-LH1 supercomplex.

	WT monomer	WT dimer	Δ*pufY* monomer	Δ*pufY* dimer	Δ*pufX*	Δ*pufXY*
τ_{EET} / ps	41 (1)	41 (1)	44 (1)	39 (1)	49 (2)	59 (2)
$FWHM_{EET}$ / ps	64 (2)	60 (3)	65 (4)	59 (3)	84 (6)	109 (8)

7. Analysis of kinetic processes with broad distributions

Lifetime distributions obtained from LDA are particular sensitive to noise within the dataset, with even minimal noise levels introducing significant artificial oscillating pre-exponential amplitudes.[8,9] Regularization is an important procedure within LDA in order to penalize large amplitude coefficients within the fitting process, effectively smoothing the data. Tikhonov regularization, as employed in OPTIMUS, can lead to broadening of kinetic distributions, which is exacerbated by increasing noise levels within the TA dataset.[8] To explore whether the broader distributions of lifetimes observed for RC ← LH1 EET of the Δ*pufXY* and Δ*pufX* supercomplexes is a result of regularization within the LDA fit, GLA of the TA data was performed.

Initially, a three-compartment parallel scheme was employed, the initial lifetime guesses were taken from the two largest peaks in the LDKT and a third lifetime to deal with species decaying beyond the timeframe probed. The decay associated difference spectra (DADS) resulting from this analysis for t_1 and t_2 resemble the LADS assigned to EEA and EET, with very similar lifetimes, from the LDA. t_3 decays beyond the experimental window, as such no relevance can be interpreted from the fitted lifetime, and contains features assignable to the decay of RC features. The results of these fits to the 900 nm kinetic trace is shown in Figure S17. A good fit is observed for the monomeric and dimeric WT and Δ*pufY* RC-LH1 supercomplexes, however a poor fit around 50-200 ps is observed for the Δ*pufX* and Δ*pufXY* species.

A 4-compartment parallel scheme was then employed, the initial lifetime guesses for the shortest (t_1) and longest (t_4) lifetime compartments were chosen from the results of the 3-compartment fit, however, those for compartments t_2 and t_3 were chosen from the half-maximum of the EET lifetime distribution observed in Figure 4 a and b for each RC-LH1 supercomplex. The DADS resulting from this analysis for t_2 and t_3 are remarkably similar in appearance to both one another, and that obtained in the 3-compartment fit. A small improvement of this 4-compartment fit is observed to the data at 900 nm for the monomeric and dimeric WT and Δ*pufY* RC-LH1 supercomplexes (as may be expected from inclusion of an additional fitting parameter), however, a notable improvement to the fit between 50-200 ps is now observed Δ*pufX* and Δ*pufXY* species, Figure S17. Consistent with the increased distribution of EET lifetimes observed in the LDKT, the difference between t_2 and t_3 is significantly larger for the Δ*pufX* and Δ*pufXY* RC-LH1 supercomplexes. Although the resulting decay-associated spectra contain spectral signatures of chromophores of interest, and hence are often assigned to specific photophysical processes, it is important to acknowledge that the fitting results are simply a mathematical model of the data, and they do not necessarily represent a physical kinetic model. These observations (the similarity of the DADS associated with t_2 and t_3 in the 4-compartment GLA fit) and the considerable improvement in fit upon inclusion of a second decay function representing the EET process for the Δ*pufX* and Δ*pufXY* species is consistent with an increased EET distribution in these RC-LH1 supercomplex. However, a direct comparison of EET lifetimes across the range of RC-LH1 supercomplexes studied cannot be easily made from such analysis. Our aim is not to fully describe the complex kinetics observed for these RC-LH1 supercomplexes using GLA, however, we note that dispersive kinetics can be modelled through the use of stretched exponential functions within GLA,[6] and, to the best of our knowledge, there is no publicly available software to perform these fits.

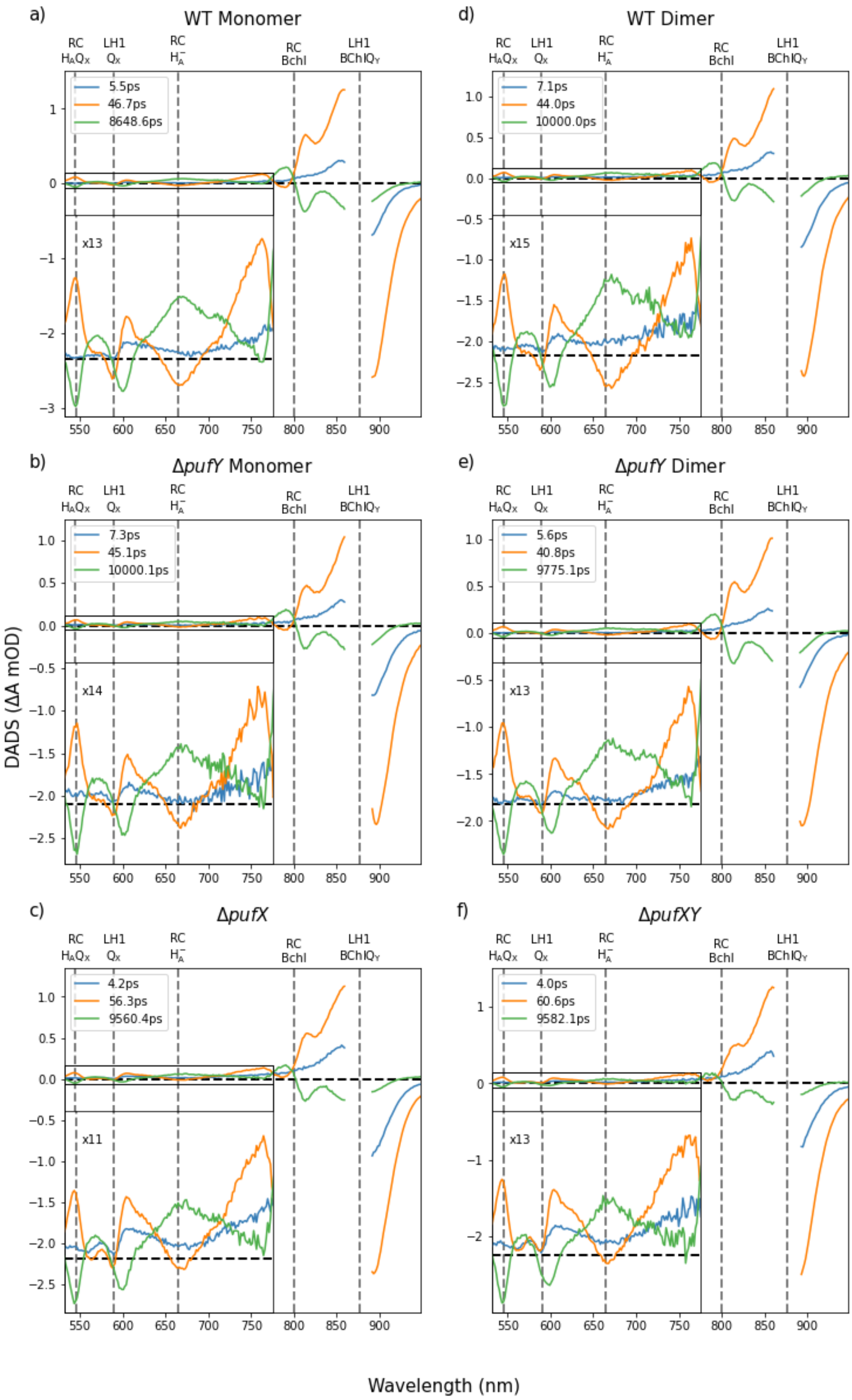

Figure S15. DADs generated from performing a 3-compartment parallel GLA with the initial guess coming from peaks found in LDKT for RC-LH1 complexes, a) WT monomer, b) Δ*pufY* monomer, c) Δ*pufX*, d) WT dimer, e) Δ*pufY* dimer, and f) Δ*pufXY*. Lifetimes resulting from the fit are indicated within the legend.

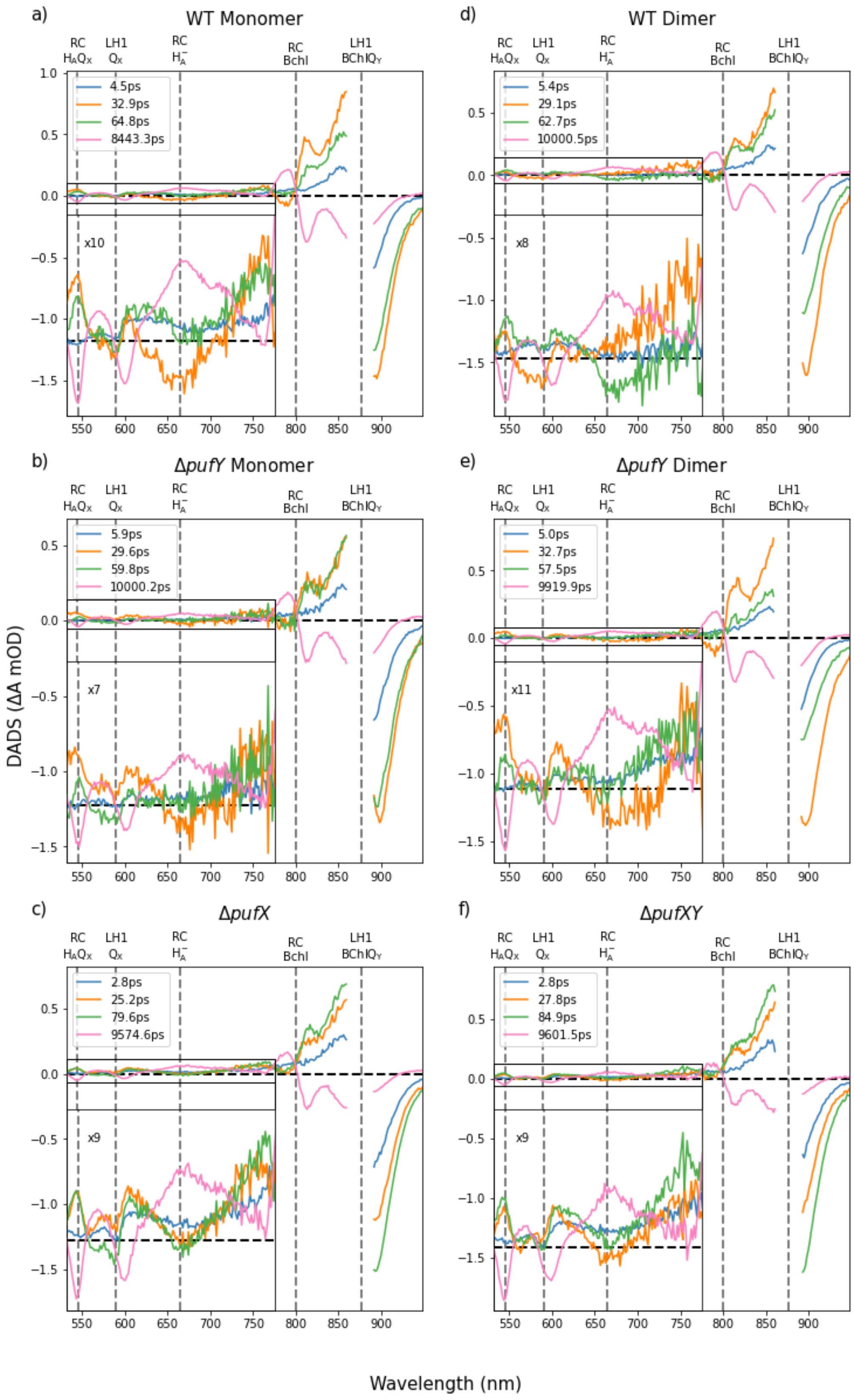

Figure S16. DADs generated from performing a 4 compartment parallel GLA with initial guess of t1 coming from peak found in LDA while t2 and t3 our set to be either side of the main LDA peak assigned to EET with t4 set to 10000ps to deal with residual signal of TA data for RC-LH1 complexes, a) WT monomer, b) Δ*pufY* monomer, c) Δ*pufX*, d) WT dimer, e) Δ*pufY* dimer, and f) Δ*pufXY*. Lifetimes resulting from the fit are indicated within the legend.

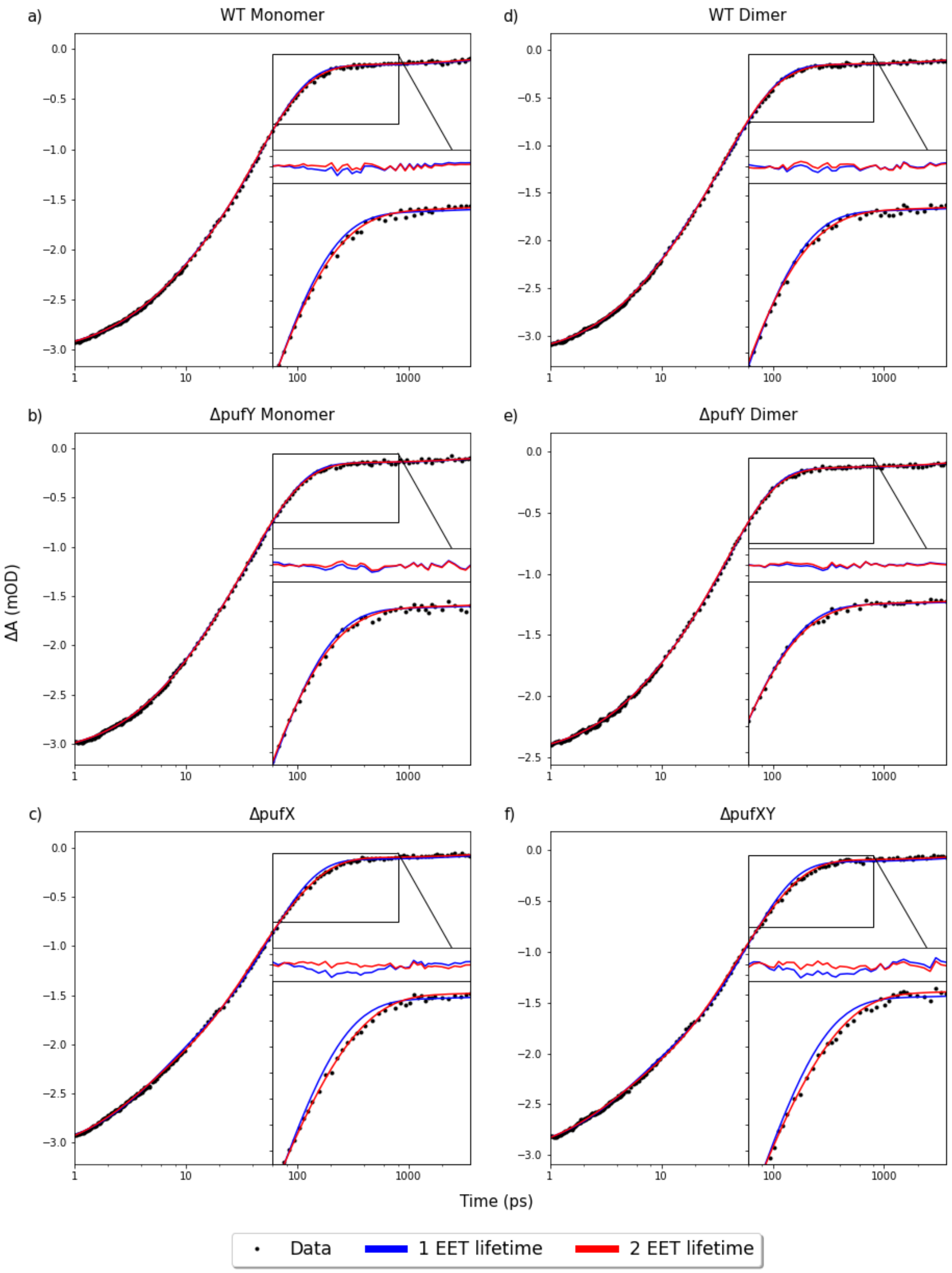

Figure S17. Fits to the kinetics observed at 900nm of the raw data (black dots), from 3-compartment GLA with single EET lifetime (blue) and 4-compartment GLA with two EET lifetimes (red) [see text] for a) WT monomer, b) Δ*pufY* monomer, c) Δ*pufX*, d) WT dimer, e) Δ*pufY* dimer, and f) Δ*pufXY*. A magnified view of the 50 - 500 ps timescale, along with the associated residuals of the fits, are shown in the insets.

8. LH1 peak shift

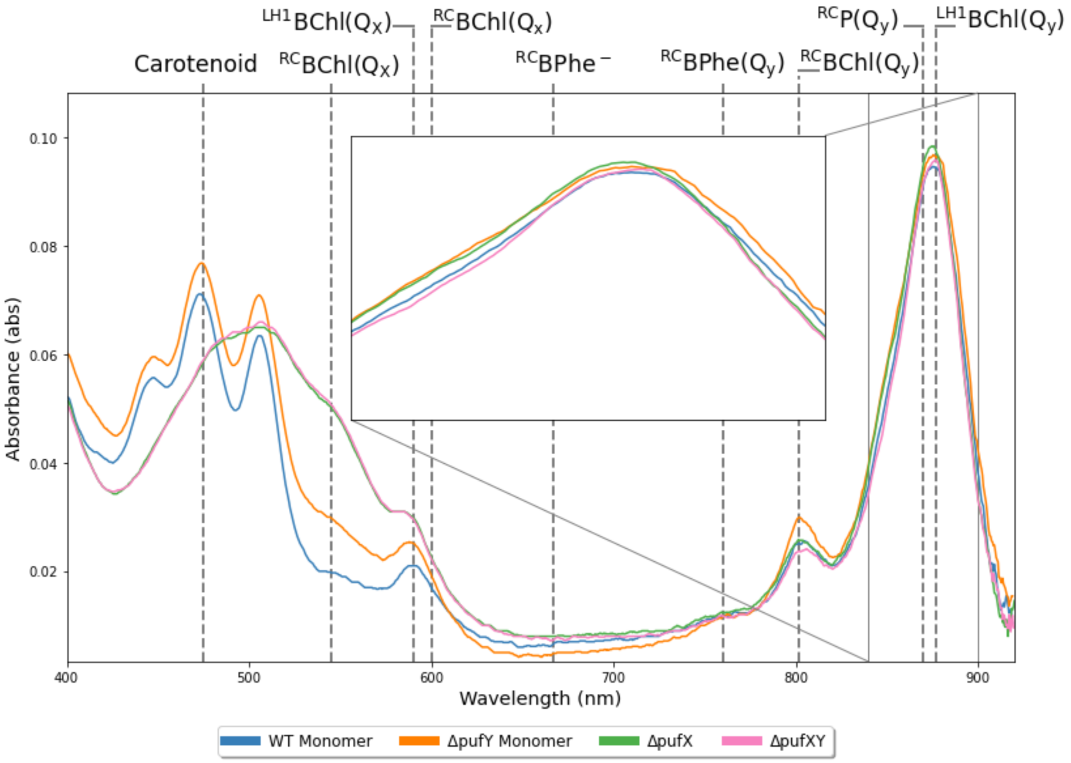

Figure S18. UV/Vis spectra of the WT monomer, *ΔpufY* monomer, *ΔpufXY* monomer and *ΔpufX* monomer as indicated. The inset shows a zoom of the ^{LH1}BChl(Q_Y) absorption peak. Note that the Δ*pufX* and Δ*pufXY* strains were grown microoxically in the dark, given their inability to photosynthesize. The Δ*pufX* and Δ*pufXY* RC–LH1 complexes have a broad absorption and maxima at 481, 509 and 545 nm, and thus a visibly different color, because of the conversion of the carotenoid spheroidene to spheroidenone under the microoxic culture conditions.

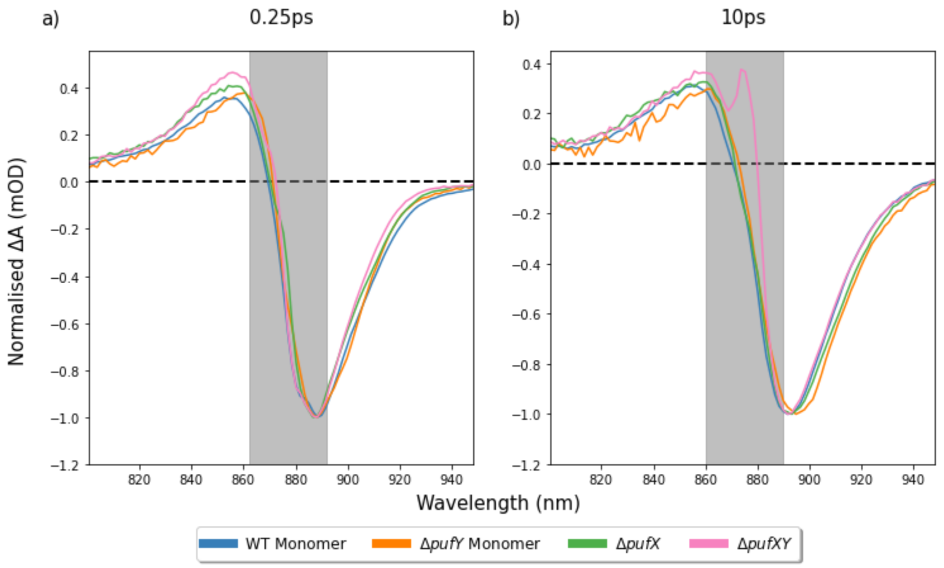

Figure S19. TA spectra of the WT monomer, *ΔpufY* monomer, *ΔpufXY* monomer and *ΔpufX* monomer as indicated at a pump-probe delay of a) 0.25 ps and b) 10 ps.

9. 900 nm Kinetic Trace

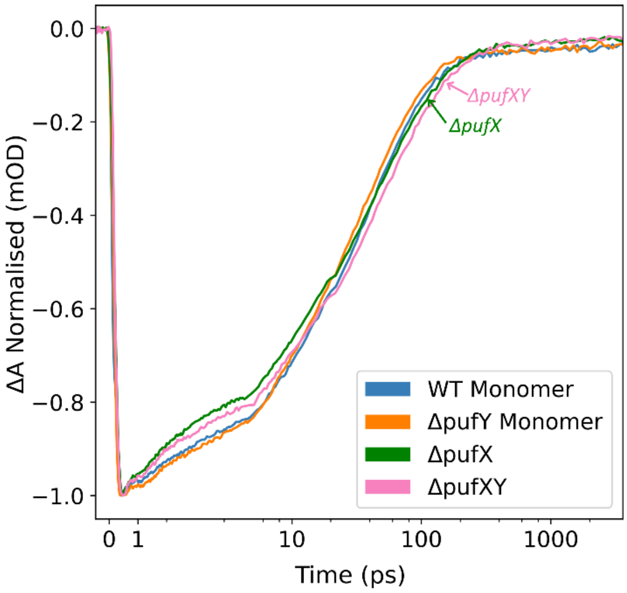

Figure S20. Kinetic trace at 900nm normalised to peak minima of the WT monomer, *ΔpufY* monomer, *ΔpufX* monomer and *ΔpufXY* monomer. The x-axis is linear up to 5 ps then logarithmic beyond

10. Structural Analysis

Table S2. Comparison of the distances BChl(Mg)–BChl(Mg) in LH1, and between the RC-LH1 special pairs of *Rba. Sphaeroides* RC-LH1 variants studied.

RC-LH1	LH1-LH1 (Å)		RC-LH1 (Å)
	Intra	Inter	
Rba. sphaeroides WT (mono)	9.4 ± 0.1	8.6 ± 0.1	45.2 ± 3.8
Rba. sphaeroides WT (dimer)	9.3 ± 0.2	8.5 ± 0.1	44.3 ± 3.7
*Rba. sphaeroides ΔpufY (*mono)	9.9 ± 0.3	8.0 ± 0.2	44.3 ± 2.8
Rba. sphaeroides ΔpufY (dimer)	9.8 ± 0.3	8.2 ± 0.2	43.7 ± 3.0
Rba. sphaeroides ΔpufX	9.9 ± 0.1	8.0 ± 0.1	46.4 ± 3.7

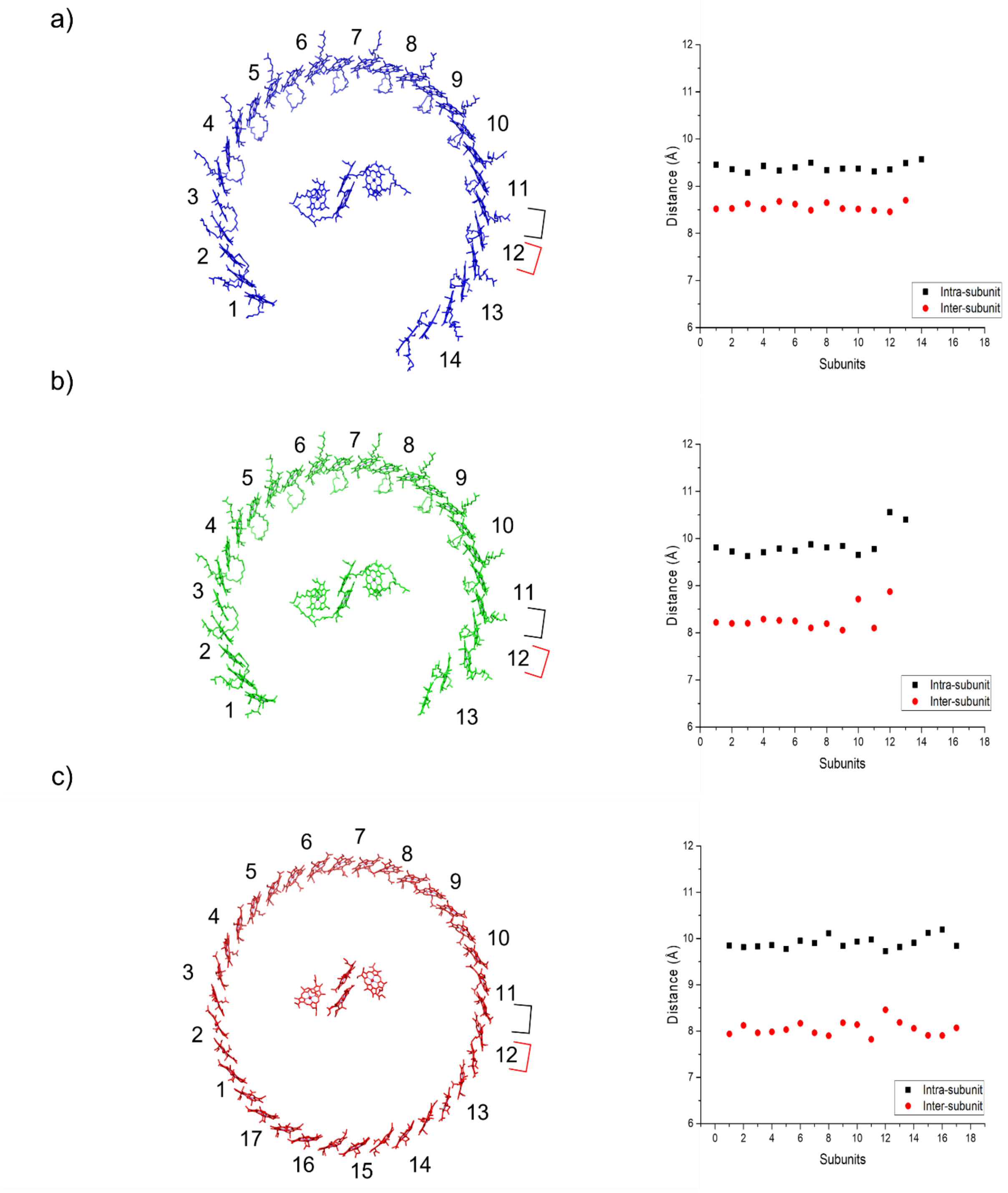

Figure S21. Intra- and inter-subunit BChl arrangement within LH1 (right) and measured intra- and inter-subunit Mg-Mg distances between the BChls within LH1 (left) on (a) WT monomer, (b) Δ*pufY* monomer, (c) Δ*pufX* RC-LH1 monomer

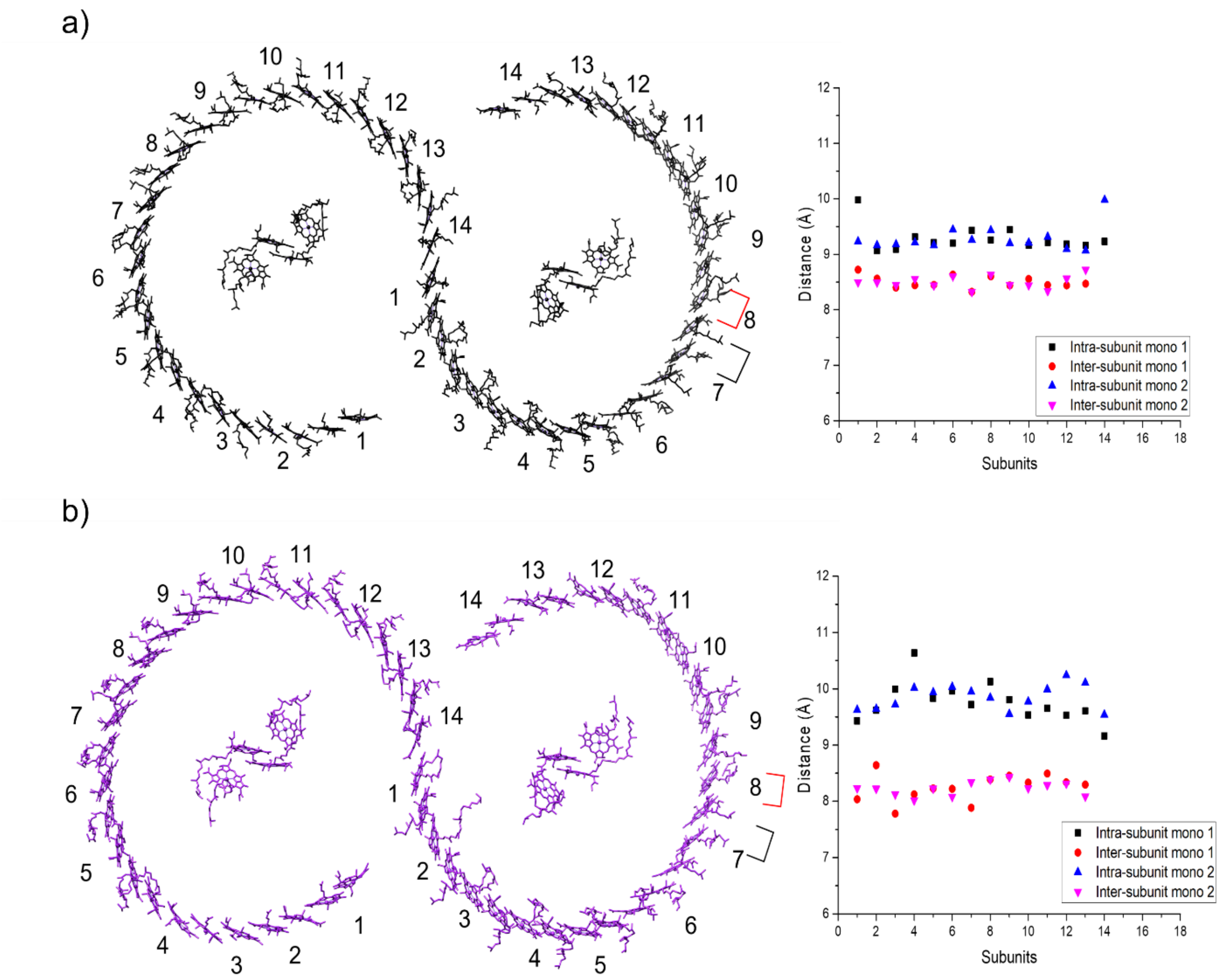

Figure S22. Intra- and inter-subunit BChl arrangement within LH1 (right) and measured intra- and inter-subunit Mg-Mg distances between the BChls within LH1 (left) on a) WT RC-LH1 dimer b) Δ*pufY* RC-LH1 dimer.

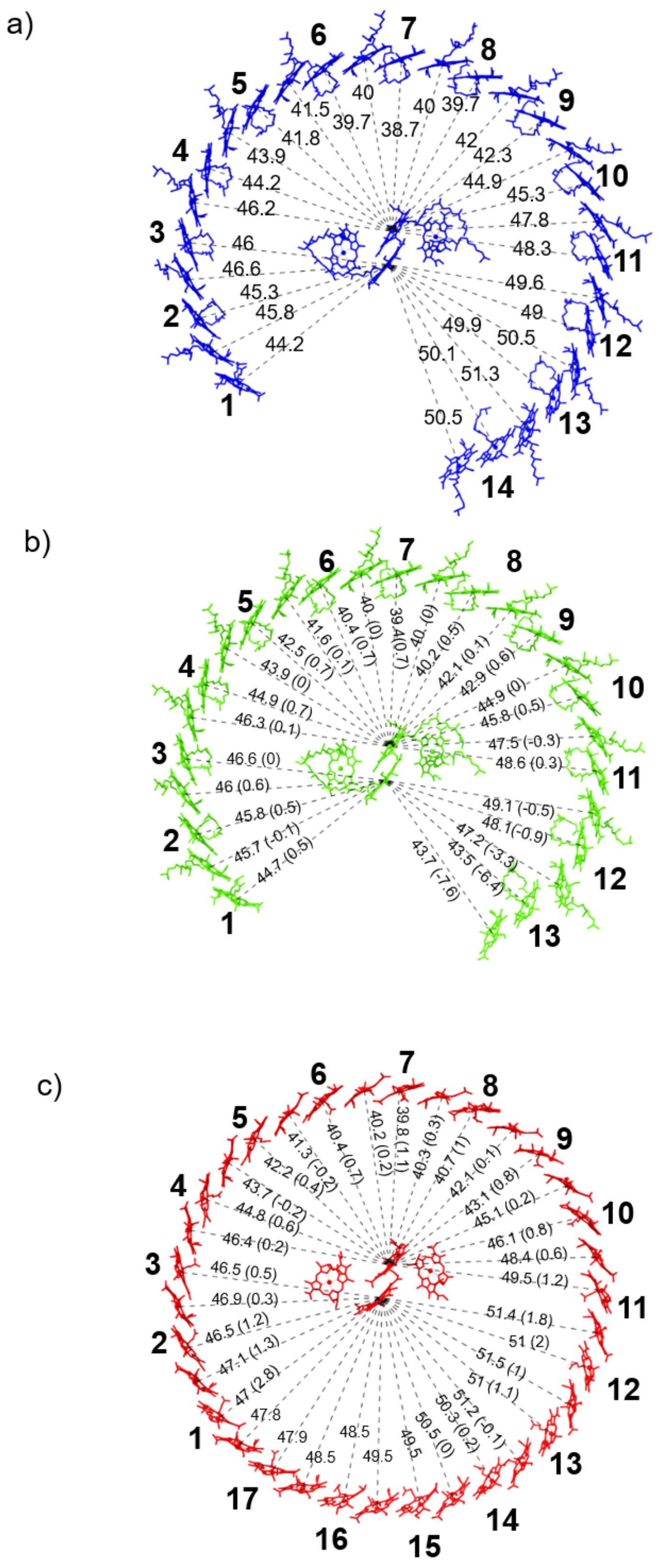

Figure S23. Distances between LH1 BChl *a* to the closest BChl *a* of the special pair in the RC of: (a) WT monomer, (b) *ΔpufY* monomer, and c) *ΔpufX* monomer. Measurements in bracket indicates difference to the WT monomer

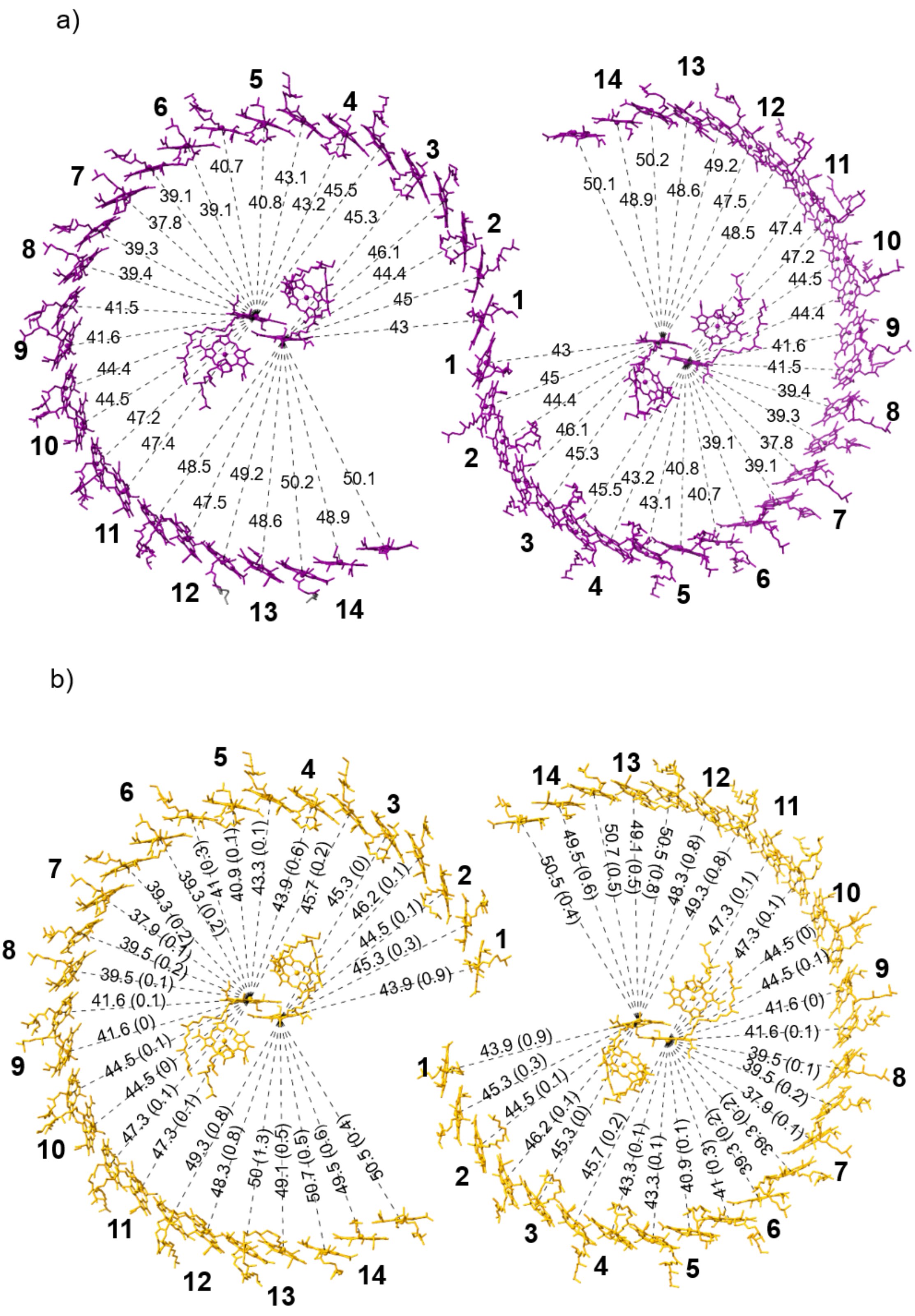

Figure S24. Distances between LH1 BChl *a* to the closest BChl *a* of the special pair in the RC of: (a) WT dimer type 1, and (b) WT dimer type 2. Measurement in bracket indicates difference to the WT dimer type 1.

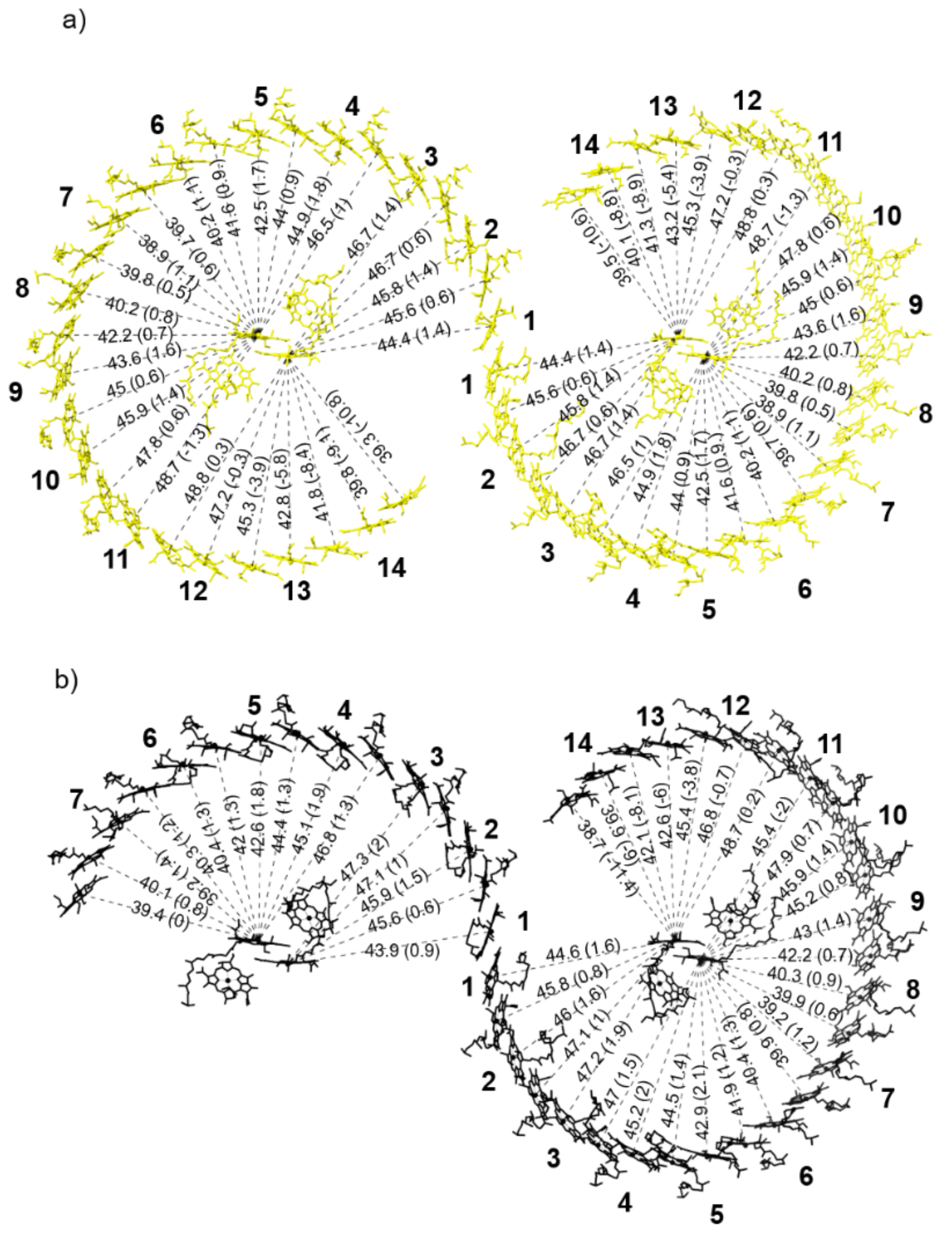

Figure S25. Distances between LH1 BChl *a* to the closest BChl *a* of the special pair in the RC of: (a) *ΔpufY* dimer type 1 , and (b) *ΔpufY* dimer type 2. Measurement in bracket indicates difference to the WT dimer type 1.

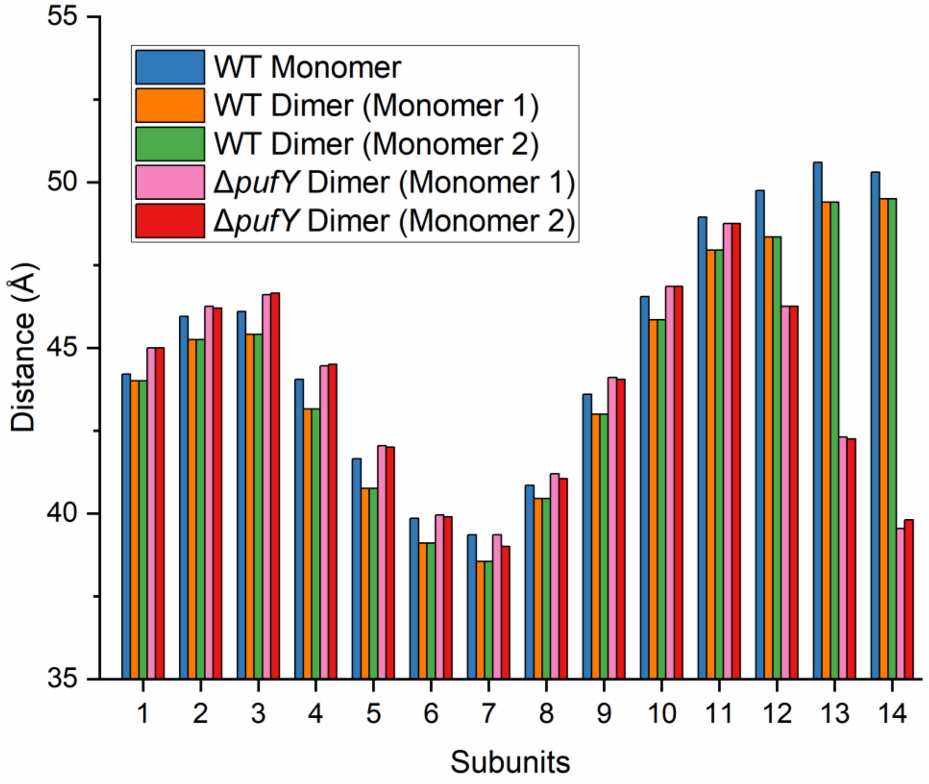

Figure S26. Average distances (measured between Mg ions) between each LH1 BChl *a* to the closest BChl *a* of the special pair of the WT monomer, WT dimer type 1 and *ΔpufY* dimer type 1. The measurement for each BChl in LH1 subunit are shown in Figures S23 and S24.

Supplementary references

(1) Cao, P.; Bracun, L.; Yamagata, A.; Christianson, B. M.; Negami, T.; Zou, B.; Terada, T.; Canniffe, D. P.; Shirouzu, M.; Li, M.; Liu, L. N. Structural Basis for the Assembly and Quinone Transport Mechanisms of the Dimeric Photosynthetic RC–LH1 Supercomplex. *Nat Commun* **2022**, *13* (1), 1–12. https://doi.org/10.1038/s41467-022-29563-3.

(2) Forster, M.; Cheung, D. W. F.; Gardner, A. M.; Cowan, A. J. Potential and Pitfalls: On the Use of Transient Absorption Spectroscopy for in Situ and Operando Studies of Photoelectrodes. *Journal of Chemical Physics* **2020**, *153* (15), 4–6. https://doi.org/10.1063/5.0022138.

(3) Ruckebusch, C.; Sliwa, M.; Pernot, P.; de Juan, A.; Tauler, R. Comprehensive Data Analysis of Femtosecond Transient Absorption Spectra: A Review. *Journal of Photochemistry and Photobiology C: Photochemistry Reviews* **2012**, *13* (1), 1–27. https://doi.org/10.1016/j.jphotochemrev.2011.10.002.

(4) Van Stokkum, I. H. M.; Larsen, D. S.; Van Grondelle, R. Global and Target Analysis of Time-Resolved Spectra. *Biochim Biophys Acta Bioenerg* **2004**, *1657* (2–3), 82–104. https://doi.org/10.1016/j.bbabio.2004.04.011.

(5) Slavov, C.; Hartmann, H.; Wachtveitl, J. Implementation and Evaluation of Data Analysis Strategies for Time-Resolved Optical Spectroscopy. *Anal Chem* **2015**, *87* (4), 2328–2336. https://doi.org/10.1021/ac504348h.

(6) Slavov, C.; Fischer, T.; Barnoy, A.; Shin, H.; Rao, A. G.; Wiebeler, C.; Zeng, X.; Sun, Y.; Xu, Q.; Gutt, A.; Zhao, K. H.; Gärtner, W.; Yang, X.; Schapiro, I.; Wachtveitl, J. The Interplay between Chromophore and Protein Determines the Extended Excited State Dynamics in a Single-Domain Phytochrome. *Proc Natl Acad Sci U S A* **2020**, *117* (28), 16356–16362. https://doi.org/10.1073/pnas.1921706117.

(7) Bonneau, R.; Wirz, J.; Zuberbuhler, A. D. Methods for the Analysis of Transient Absorbance Data (Technical Report). *Pure and Applied Chemistry* **1997**, *69* (5), 980–992. https://doi.org/10.1351/pac199769050979.

(8) Slavov, C.; Hartmann, H.; Wachtveitl, J. Implementation and Evaluation of Data Analysis Strategies for Time-Resolved Optical Spectroscopy. *Anal Chem* **2015**, *87* (4), 2328–2336. https://doi.org/10.1021/ac504348h.

(9) Croce, R.; Müller, M. G.; Bassi, R.; Holzwarth, A. R. Carotenoid-to-Chlorophyll Energy Transfer in Recombinant Major Light-Harvesting Complex (LHCII) of Higher Plants. I. Femtosecond Transient Absorption Measurements. *Biophys J* **2001**, *80* (2), 901–915. https://doi.org/10.1016/S0006-3495(01)76069-9.
